# Maternal vaginal microbiome composition does not affect development of the infant gut microbiome in early life

**DOI:** 10.3389/fcimb.2023.1144254

**Published:** 2023-03-30

**Authors:** Scott J. Dos Santos, Zahra Pakzad, Arianne Y. K. Albert, Chelsea N. Elwood, Kirsten Grabowska, Matthew G. Links, Jennifer A. Hutcheon, Evelyn J. Maan, Amee R. Manges, Tim J. Dumonceaux, Zoë G. Hodgson, Janet Lyons, Sheona M. Mitchell-Foster, Soren Gantt, K.S. Joseph, Julie E. Van Schalkwyk, Janet E. Hill, Deborah M. Money

**Affiliations:** ^1^ Department of Veterinary Microbiology, Western College of Veterinary Medicine, University of Saskatchewan, Saskatoon, SK, Canada; ^2^ Department of Microbiology and Immunology, Faculty of Science, University of British Columbia, Vancouver, BC, Canada; ^3^ Women’s Health Research Institute, B.C. Women's Hopsital, Vancouver, BC, Canada; ^4^ Department of Obstetrics and Gynaecology, Faculty of Medicine, University of British Columbia, Vancouver, BC, Canada; ^5^ Department of Animal and Poultry Science, University of Saskatchewan, Saskatoon, SK, Canada; ^6^ Department of Computer Science, University of Saskatchewan, Saskatoon, SK, Canada; ^7^ School of Population and Public Health, University of British Columbia, Vancouver, BC, Canada; ^8^ British Columbia Centre for Disease Control, Vancouver, BC, Canada; ^9^ Agriculture and Agri-Food Canada, Saskatoon, SK, Canada; ^10^ Midwifery Program, Faculty of Medicine, University of British Columbia, Vancouver, BC, Canada; ^11^ Centre de Recherche du CHU Sainte-Justine, Montréal, QC, Canada

**Keywords:** vaginal microbiome, infant stool microbiome, infant gut, *cpn*60, vaginal seeding, birth mode, microbiome

## Abstract

Birth mode has been implicated as a major factor influencing neonatal gut microbiome development, and it has been assumed that lack of exposure to the maternal vaginal microbiome is responsible for gut dysbiosis among caesarean-delivered infants. Consequently, practices to correct dysbiotic gut microbiomes, such as vaginal seeding, have arisen while the effect of the maternal vaginal microbiome on that of the infant gut remains unknown. We conducted a longitudinal, prospective cohort study of 621 Canadian pregnant women and their newborn infants and collected pre-delivery maternal vaginal swabs and infant stool samples at 10-days and 3-months of life. Using *cpn*60-based amplicon sequencing, we defined vaginal and stool microbiome profiles and evaluated the effect of maternal vaginal microbiome composition and various clinical variables on the development of the infant stool microbiome. Infant stool microbiomes showed significant differences in composition by delivery mode at 10-days postpartum; however, this effect could not be explained by maternal vaginal microbiome composition and was vastly reduced by 3 months. Vaginal microbiome clusters were distributed across infant stool clusters in proportion to their frequency in the overall maternal population, indicating independence of the two communities. Intrapartum antibiotic administration was identified as a confounder of infant stool microbiome differences and was associated with lower abundances of *Escherichia coli*, *Bacteroides vulgatus*, *Bifidobacterium longum* and *Parabacteroides distasonis*. Our findings demonstrate that maternal vaginal microbiome composition at delivery does not affect infant stool microbiome composition and development, suggesting that practices to amend infant stool microbiome composition focus factors other than maternal vaginal microbes.

## Introduction

The human gut harbours a diverse microbial community that provides a multitude of immunological and metabolic functions linked to infant development and homeostasis (Thomas et al., 2017). Dysbiosis of the infant gut microbiome appears to be associated with adverse health outcomes in childhood including asthma, atopy, obesity, and various autoimmune diseases (Stiemsma and Michels, 2018). Precisely how the infant gut microbiome is established and what influences optimal versus dysbiotic manifestations remain a key area in health research.

Many studies have implicated mode of delivery as a major factor in the establishment of the early neonatal gut microbiome. One notable cohort study of nine infants from 2010 documented an abundance of *Lactobacillus* and other known vaginal genera in infants born vaginally, while infants delivered by caesarean section (C/S) exhibited stool microbiomes dominated by skin commensals, such as *Staphylococcus* and *Corynebacterium* (Dominguez-Bello et al., 2010). While more recent, larger studies have not identified vaginal microbes as key members of the neonatal or early infant gut microbiome, compositional differences between vaginally and C/S-delivered infants remain a consistent finding. An increased relative abundance of *Escherichia*, *Bacteroides* and *Bifidobacterium* spp. in infant stool microbiomes has been associated with vaginal birth, while increases in *Streptococcus*, *Enterococcus* and *Klebsiella* spp. have been associated with C/S delivery (Azad et al., 2016; Dominguez-Bello et al., 2016; Yassour et al., 2016; Reyman et al., 2019; Shao et al., 2019; Mitchell et al., 2020; Busi et al., 2021; Song et al., 2021; Wilson et al., 2021). Despite the substantive compositional differences between delivery mode groups in many studies, there is inconsistency surrounding the species involved and the persistence of this dysbiosis during early life (Azad et al., 2016; Chu et al., 2017; Stewart et al., 2018; Shao et al., 2019; Busi et al., 2021). Furthermore, previous studies tended not to differentiate between elective and emergency C/S, even though neonates delivered by emergency C/S may have experienced prolonged exposure to vaginal microbiota after rupture of the foetal membranes (Azad et al., 2016; Stewart et al., 2018; Shao et al., 2019).

While the link between birth mode and infant gut microbiome development is well-described, there is little data regarding the impact of the maternal vaginal microbiome despite the latter being the primary microbial niche to which most birthing neonates are exposed. There is a substantive variability in vaginal microbiome composition between women and if the vaginal microbiome is functionally ‘seeding’ or otherwise influencing the infant gut microbiome as has been suggested (Dominguez-Bello et al., 2016), an association between the different vaginal microbiome profiles of women and their infants’ gut microbiomes should exist. In addition, this relationship should not be present in elective, pre-labour caesarean births but should be detectable after vaginal delivery (and likely to some degree, after emergency caesarean delivery following active labour and/or ruptured membranes). Presently, this association has not been proven; however, practices to correct dysbiotic infant gut microbiomes based on direct transfer of ‘missing’ vaginal microbes in cases of C/S delivery have arisen, though are not recommended by obstetric and gynaecological societies due to lack of efficacy data and potential safety concerns (Cunnington et al., 2016; Dominguez-Bello et al., 2016; Wharton and Birsner, 2017; Song et al., 2021).

Given that the role of the maternal vaginal microbiome in infant gut microbiome development is understudied compared to factors such as delivery mode, breastfeeding, and infant antibiotic exposure, we aimed to investigate the effect of this microbial community on infant stool microbiomes in a large-scale, longitudinal study. Here, we used *cpn*60 microbiome profiling in a cohort of over 600 Canadian women and their newborns enrolled in the LEGACY study to determine whether the maternal vaginal microbiome composition influences or predicts that of the infant stool, and if this is affected by delivery mode as might be expected.

## Materials and methods

### Study population and data collection

Healthy, low risk, pregnant individuals delivering at term were recruited into the Maternal Microbiome LEGACY Project from three hospitals across British Columbia (BC), Canada between March 2018-March 2020: BC Women’s Hospital + Health Centre (Vancouver), Surrey Memorial Hospital (Surrey) and University Hospital of Northern BC (Prince George). These centres were chosen with the goal of enrolling a large, sociodemographically diverse, multi-ethnic study population. Participants were enrolled based on the following inclusion criteria: >18 years of age, ≥37 weeks gestational age at delivery, no known major foetal anomalies and singleton or twin gestation. Participants were excluded based on the following criteria: inability to provide informed consent, participation in drug or probiotic trials, triplet or higher order gestation, placenta previa at delivery, placental abruption, and emergency intrapartum complications that preclude time for engagement in research.

Informed written consent for study participation was obtained and ethics approval was granted by the University of British Columbia Children’s and Women’s Health Centre Research Ethics Board, harmonised with partner boards in the Fraser Health Authority and Northern Health Authority (Certificate no. H17-02253). Participants were recruited either during pregnancy or at the time of admission for delivery. Targeted recruitment of individuals with planned caesarean section and planned vaginal delivery ensured appropriate numbers of participants in the groups of interest. Target study population size was calculated to provide the best power to detect an interaction term in a multinomial regression model. Based on a maximum effect size of 0.41 from Human Microbiome Project data and a minimum of 0.2, a sample size of at least 600 would permit detection of effect sizes of at least 0.21 at power = 0.8, alpha = 0.05. Demographic and clinical data were collected by research staff *via* interview and medical chart review and entered into the Research Electronic Data Capture (REDCap) database securely hosted at BC Children’s Hospital Research Institute (Harris et al., 2019).

### Sample collection

Maternal vaginal swabs of the posterior fornix and lateral vaginal wall were collected at first presentation to the labour/delivery assessment area, by a nurse, midwife, or clinician prior to examinations and procedures associated with the labour/delivery/birth process. Neonatal meconium, defined as the first collected stool specimen within 72 hours of birth, and two infant stool samples were collected at follow-up visits at 10 days ( ± 3 days) and 3 months ( ± 2 weeks) postpartum, respectively. Diapers with stool were collected by parents and kept refrigerated in sealed bags until collection by the study team at home visits either the same day or next day. Samples were transported to the lab on ice in a cooler, and stool was scraped from diapers using sterile spatulas in a biosafety cabinet and aliquoted into cryovials for storage at -80°C. Empty cryovials were processed in the same manner as samples to generate mock sample controls for assessment of contamination introduced during the handling process. Samples were shipped from the central collection lab at BC Women’s Health Research Institute to the University of Saskatchewan on dry ice and immediately stored at -80°C until DNA extraction.

### DNA extraction, library preparation and sequencing

On the day of DNA extraction, 1 mL of sterile phosphate-buffered saline (pH 7.4) was added to thawed vaginal swab storage tubes. Following vigorous vortexing, swab fluid was transferred to sterile 1.5 mL tubes; 100 µL was taken as input for DNA extraction and the remainder was stored at -80°C. Stool was removed from cryovials using disposable sterile spatulas in a biosafety cabinet and 200 mg was transferred to sterile 2 mL tubes for DNA extraction. Total genomic DNA was extracted with a MagMax DNA Ultra 2.0 kit (Applied Biosystems, Waltham, MA, USA) on an automated KingFisher Flex platform (ThermoFisher Scientific, Waltham, MA, USA) which employs a combination of mechanical and chemical lysis. Extractions of vaginal and stool samples were performed on different days to limit cross-contamination. Mock samples (empty tubes left open during sample processing in Vancouver) and extraction-negative controls (400 µL sterile molecular biology-grade water) were included with each batch of samples to assess contamination. Kit controls were also generated by separate extraction of kit reagents only.

The universal barcode region of the *cpn*60 gene was amplified as described previously (Fernando and Hill, 2021). A mixture of 20 plasmids harbouring *cpn*60 sequences from known vaginal microbiome species (mixed vaginal panel, MVP) mixed in equal proportions was used as a positive control (Dumonceaux et al., 2009). Amplicons were purified and indexed according to the standard Illumina 16S metagenomic sequencing library preparation protocol (Illumina, 2013). Indexed amplicons were quantified using Qubit fluorometry, normalised to 4 nM and pooled. Diluted 10 pM libraries (5% PhiX) were sequenced on an Illumina MiSeq platform using 500-cycle v2 reagent kits (401 R1, 101 R2). Only R1 sequence reads were used for analysis as read-pair overlap is not possible and only 150 bp of the *cpn*60 barcode region is required for accurate taxonomic identification (Vancuren et al., 2020).

### Quality control and analysis of sequencing data

Amplification primers were removed using cutadapt (Martin, 2011) and reads were trimmed to an average Phred score of Q30 over a 4 bp window and minimum length of 150 bp using Trimmomatic (Bolger et al., 2014). Variant calling was performed in the QIIME2 (Bolyen et al., 2019) package using DADA2 (Callahan et al., 2016) to generate 150 bp amplicon sequence variants (Vancuren et al., 2020) (ASVs) and the resulting feature table was converted to tsv format. Taxonomy was assigned by comparison to a non-redundant version of cpnDB (cpndb_nr_20210316) using wateredBLAST, and ASVs with <55% sequence similarity to a cpnDB hit were discarded (Schellenberg et al., 2009; Vancuren and Hill, 2019). ASVs aligning to the same cpnDB entry were collapsed into ‘nearest neighbours’ and their frequencies combined.

Feature tables were screened for potential contaminants in RStudio v.1.4.1717 [R v4.1.1 (R Core Team, 2022)] using the decontam (Davis et al., 2018) package. Any infant stool microbiomes with <1,000 reads were excluded from downstream analysis. Heatmaps were built from proportional abundance data of vaginal and stool microbiome profiles: all taxa were used for hierarchical clustering of samples (Ward linkage) based on Euclidean distance matrices calculated by the vegan (Oksanen et al., 2020) package. Clusters within vaginal, 10-day stool and 3-month stool microbiome profile datasets were defined algorithmically by silhouette indices using the NbClust (Charrad et al., 2014) package, and were bootstrapped to assess cluster support using the fpc (Hennig, 2007) package. Independence of maternal and infant microbiome clusters was examined using correspondence analysis (implemented by the FactoMineR (Lê et al., 2008) package) and Fisher’s exact test. Feature tables containing read counts for vaginal and stool samples underwent centre log-ratio transformation using the ALDEx2 (Fernandes et al., 2013) package prior to principal component analysis (PCA) and assessment of significant compositional differences using pairwise PERMANOVA (Anderson and Walsh, 2013). The aldex wrapper function was used to define differentially abundant taxa, while multivariable association analysis was conducted using the MaAsLin2 (Mallick et al., 2021) package. The Shannon-Weaver diversity index (H’) was calculated for 10-day and 3-month stool samples rarefied to 1,000 reads using the vegan (Oksanen et al., 2020) package, with differences between intrapartum antibiotic exposure and delivery mode groups investigated using Mann-Whitney U and Kruskal-Wallis tests in GraphPad Prism v9.3.1 Significant differences in the proportional abundance of differentially abundant infant stool taxa between antibiotic exposure groups were also investigated by Mann-Whitney U tests in GraphPad Prism v9.3.1, correcting for multiple testing by controlling the false discovery rate at <1%.

### Data availability

All sequence data associated with this study were deposited in the NCBI Sequence Read Archive (BioProject PRJNA824125). Annotated R code describing reproducible analysis of sequencing data, processed feature tables containing nearest neighbour read counts, and microbiome cluster metadata are available to download from the online version of this manuscript (**Supplementary Files S1–S3**).

## Results

### Clinical cohort and final sequencing dataset

We enrolled 623 participants and their infants into the LEGACY study: 247 (39.6%) participants delivered vaginally, 221 (35.5%) had an elective C/S and 155 (24.9%) had an emergency C/S (**Supplementary Table S1**), proportionally determined by our recruitment strategy. Mean age at delivery was 34.6 years, which is representative of our study hospitals’ delivery populations. Most participants self-identified as White (54.7%), 21.2% as Asian, 8.3% as South Asian, with lower percentages of other ethnicities. Twenty-one percent (21.3%) of participants tested positive for group B *Streptococcus* (GBS) on screening; 10.4% of participants had hypertension during pregnancy, and 16.5% were diagnosed with gestational diabetes (half of whom were treated with insulin). All participants undergoing elective C/S received intravenous antibiotic prophylaxis- usually cefazolin- approximately 1 hour prior to the procedure so that therapeutic concentrations are present in tissues at the time of skin incision (van Schalkwyk and Van Eyk, 2017) and those with GBS colonisation who were planning a vaginal delivery received intrapartum prophylaxis. The rate of prolonged duration of ruptured membranes (‗18 hours) was comparable between the vaginal and emergency C/S groups (9.3 vs 9.0%, respectively).

We profiled the microbiomes of 623 maternal vaginal swabs taken on admission to labour and delivery, 142 meconium samples, 581 stool samples from 10-day-old infants and 462 stool samples from 3-month-old infants. Data for LEGACY meconium samples have been described elsewhere, demonstrating lack of a detectable microbiome signature that could be differentiated from background signal and no evidence of an *in-utero* stool microbiome (Dos Santos et al., 2021). Following quality control, 71,826,602 reads were retained from 621 vaginal microbiomes, 570 10-day stool microbiomes, 460 3-month stool microbiomes and 176 controls. Complete microbiome data was available for 442 dyads (i.e., vaginal and both stool microbiome profiles). In line with recent recommendations for microbiome studies (Eisenhofer et al., 2019), a complete list of all taxa found in negative controls is shown in **Supplementary File S3**. Cross-contamination of negative controls with common vaginal and stool organisms was prevalent, with species such as *Lactobacillus crispatus* and *Escherichia coli* detected in many controls (**Supplementary File S3**). Exogenous contaminants were also apparent: *Pseudomonas tolaasii*, an environmental species, was detected in 631 samples and 89 controls and was particularly associated with certain sequencing batches. Moreover, decontam identified this species as a potential contaminant: approximately 20% of all *P. tolaasii* reads were detected in negative controls- the highest proportion of any taxon in the dataset. Accordingly, all reads aligning to this species were removed from the dataset prior to further analyses. Nineteen of twenty positive control taxa were detected across all sequencing runs, the exception being *Streptococcus macedonius*, which was not present in the plasmid library in the second batch of positive controls used in the study. With this exception, positive control composition was consistent across sequencing batches, indicating the consistency of PCR amplification.

### Maternal vaginal microbiome composition

Vaginal microbiomes were dominated by either one of several species of *Lactobacillus*, a single anaerobic species commonly associated with the vaginal environment, or were composed of a mixture of various anaerobes (**Supplementary Figure S1**). The four *Lactobacillus*-dominated community state types (CSTs) commonly reported in studies of the vaginal microbiome were well represented. In accordance with other studies of pregnant individuals (Albert et al., 2015; Freitas et al., 2017; Freitas et al., 2018; Wells et al., 2020), profiles dominated by *L. crispatus* (CST I) were the single most abundant vaginal cluster (*n* = 263), followed closely by those dominated by various or mixed obligate anaerobes (CST IV; *n* = 241). Vaginal microbiomes dominated by *L. gasseri* (CST II; *n* = 37), *L. iners* (CST III; *n* = 45) and *L. jensenii* (CST V; *n* = 37) were markedly less common. Hierarchical clustering identified a total of 16 ‘cluster types’: the four commonly reported *Lactobacillus* CSTs above, plus a further 12 that divided CST IV into 12 separate clusters. Eleven of these represent a single dominant species (such as *Megasphaera*, *Gardnerella* and *Bifidobacterium* spp.), while the twelfth was comprised of vaginal microbiomes with mixed anaerobic populations. The distribution of maternal vaginal community state types was not significantly different between women undergoing rupture of membranes before or after sample collection (*P* = 0.238, Fisher’s exact test).

### Infant stool microbiome composition

**Figure 1:**
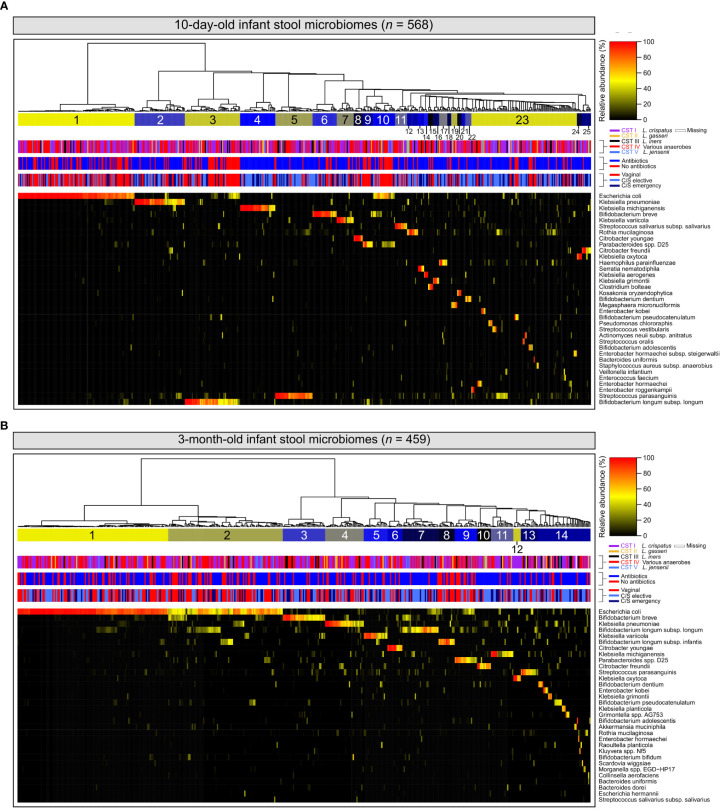
Infant stool microbiomes exhibit low diversity and are typically dominated by a single species: Hierarchical clustering of *cpn*60 stool microbiomes constructed from Euclidean distance matrices of proportional abundance data from 10-day **(A)** and 3-month **(B)** -old infants. The 35 most prevalent taxa are shown. From top to bottom, colour bars represent: infant stool microbiome clusters defined by nbClust, maternal vaginal community state type, intrapartum antibiotic exposure, and delivery mode.

Among 10-day-old infants, diversity within stool microbiomes was remarkably low and most stool microbiomes were dominated by a single species (‗80% relative abundance). Enteric taxa often reported in gut microbiome studies were commonly detected, such as *E. coli* and various members of the genera *Bifidobacterium*, *Enterococcus*, *Klebsiella*, *Bacteroides* and *Streptococcus* (Yatsunenko et al., 2012; Shao et al., 2019) (**Figure 1A**). Stool samples clustered into 25 separate clusters, with 23 defined by a single dominant species, one defined by a mixture of *E. coli*, *Bifidobacterium breve* and *Parabacteroides distasonis*, and a final cluster of microbiomes typically composed of multiple species. The latter also included small sub-clusters of several samples where a single species was dominant (e.g., *Bifidobacterium pseudocatenulatum* or *Enterobacter kobei*).

**Figure 2:**
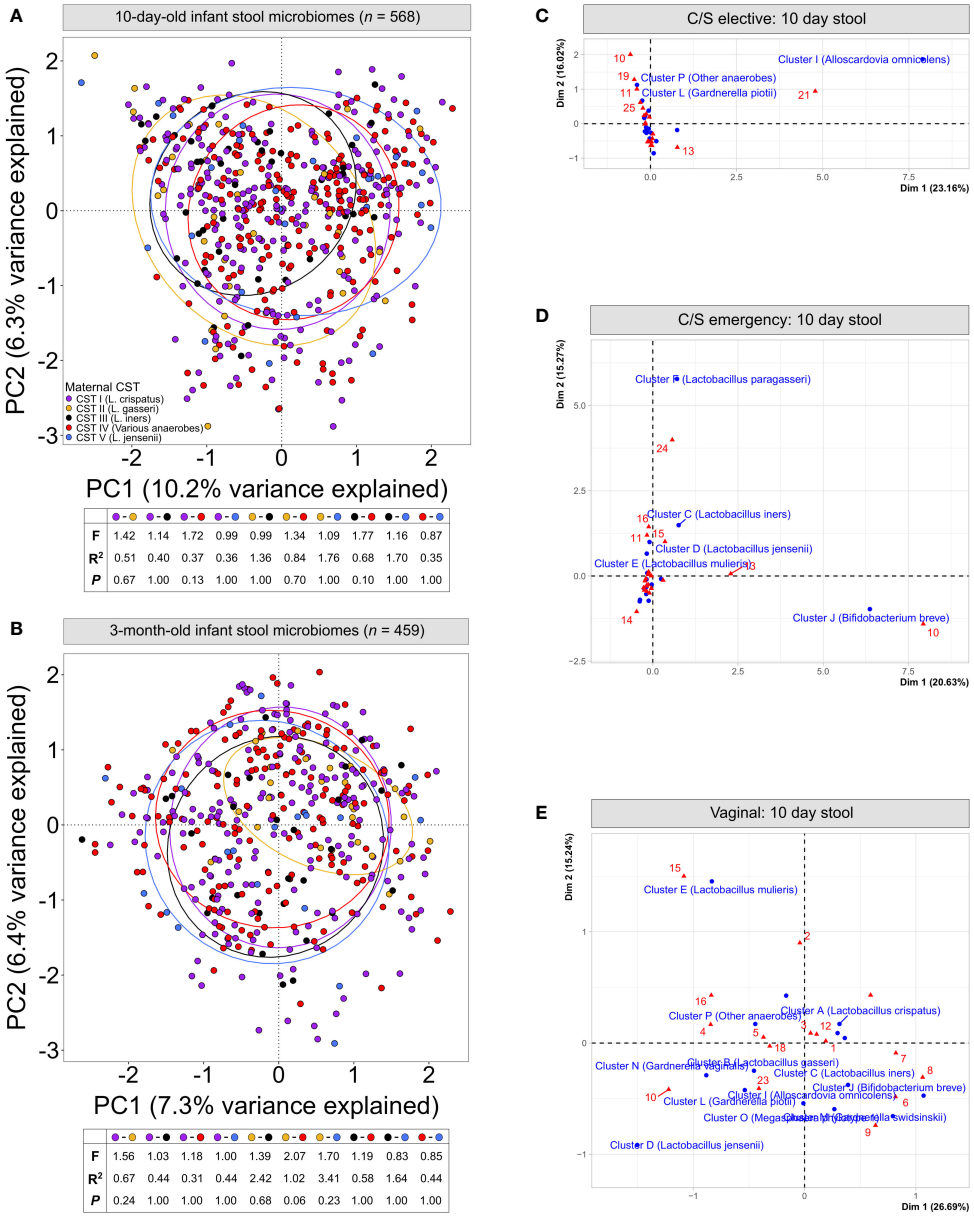
Infant stool microbiomes show no distinct clustering based on maternal vaginal CST: Principal component analysis of cpn60 stool microbiome profiles from 10-day **(A)** and 3-month **(B)** -old infants grouped by canonical maternal vaginal CSTs. No significant clustering was observed at 10-days (pairwise PERMANOVA, F = ¾1.77, R^2^ = ¾1.76, *P* = ‗0.13) or 3-months (F = ¾2.07, R^2^ = ¾3.41, *P* = ‗0.06). **(C–E)** Correspondence analysis of maternal vaginal CSTs (16 clusters) and infant stool clusters from 10-day-old infants, grouped by elective caesarean **(C)**, emergency caesarean **(D)** or vaginal delivery **(E)**.

At 3 months, the number of microbiome clusters defined decreased to 14, with 12 defined by a single species comprising at least half of the microbiome by proportion (**Figure 1B**). A further cluster was defined by a combination of *E. coli* and several species of *Bifidobacterium*, with a final cluster of mixed microbiome profiles, containing several small sub-clusters dominated by various species. Infant stool microbiomes also exhibited a significant increase in alpha diversity (Shannon-Weaver diversity index, **Figure 2A**; *P <*0.0001), with far more samples composed of multiple species found together at more even relative abundances.

At both 10 days and 3 months of life, clusters dominated by *E. coli* represented the largest number of samples of any single cluster (*n* = 116 (20.4%) and 121 (26.3%), respectively). When considering all clusters for which *E. coli* was a dominant species, this increased to 138 (24.3%) and 213 (46.4%), respectively. *Bifidobacterium* spp.– widely recognised as keystone infant gut species (Gotoh et al., 2019)- and various species of *Klebsiella* were also prevalent in infant gut microbiome profiles at both timepoints. Clusters defined by these genera accounted for 111 (19.5%) and 117 (20.5%) samples from 10-day-old infants, and 168 (36.5%) and 74 (16.0%) samples from 3-month-old infants, respectively. Relative abundances of *E. coli* and *Bifidobacterium* spp. were significantly higher at 3 months of life (**Supplementary Figure 2B** left & middle; *P <*0.0001 and <0.001 respectively), while no difference was observed for *Klebsiella* spp. (**Supplementary Figure S2B** right, *P* = 0.942).

### Vaginal microbiome exposure does not explain differences in infant stool microbiome composition, even among vaginally-delivered infants

**Figure 3:**
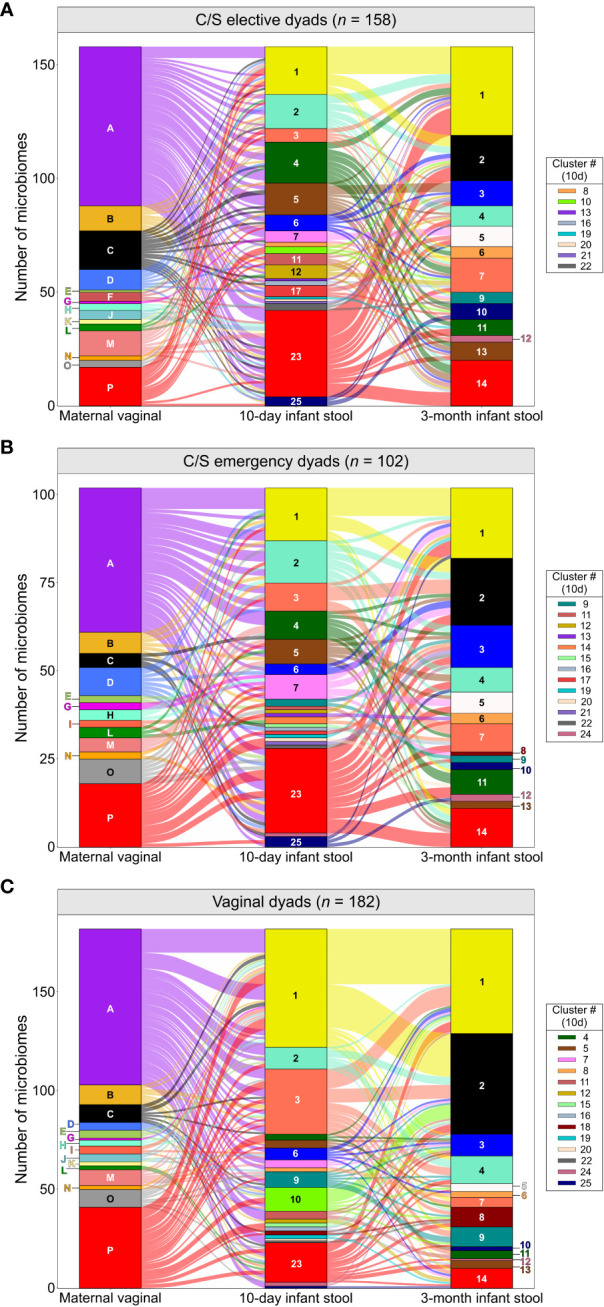
Composition of the maternal vaginal microbiome does not predict infant stool microbiome composition: Alluvial flow of mother-infant dyads from pre-delivery maternal vaginal microbiome clusters to 3-month-old infant stool microbiome clusters for infants delivered by elective C/S **(A)**, by emergency C/S **(B)** and vaginally **(C)**. Letters and numbers on stacked bars and side legend indicate microbiome clusters from **Supplementary Figure S1** and **Figure 1**. See **Supplementary Figure S9** for colour legend.

We next assessed if the maternal vaginal microbiome composition could explain differences in infant stool microbiome composition between birth modes. PCA of infant stool microbiomes showed no significant clustering of microbiome profiles at 10 days or 3 months by maternal CST (**Figures 2A, B**, *P* ‗0.13 or ‗0.24 for all comparisons, respectively). Correspondence analysis also demonstrated no evident co-clustering of maternal and infant clusters at either timepoint (**Figures 2C–E** and **Supplementary Figure S3**), and no differences were observed in the distribution of maternal vaginal microbiome clusters among infant stool microbiome clusters regardless of delivery mode (**Supplementary Figure S3D**, *P >*0.22).To investigate this further, we assessed the transition of 442 mother-infant dyads with complete profiling data between microbiome clusters over time. Regardless of birth mode, no evident patterns in the flow of microbiomes from maternal to infant clusters were discernible (**Figure 3**), with most infants in almost all 10-day clusters originating from mothers whose vaginal microbiome clusters were dominated by *L. crispatus* or various anaerobes (i.e., the two largest maternal clusters). Exceptions to this were exclusively seen in infant stool clusters comprised of a few or even single samples. Conversely, maternal vaginal clusters were distributed among stool clusters according to their frequency in the overall maternal population, indicating that the maternal vaginal microbiome holds no predictive value for infant stool microbiome composition. Lastly, we evaluated the stability of infant stool microbiomes over time and noted that over 75% of infants belonged to a cluster with a different ‘dominant’ species at 3 months compared to the 10-day sample, reflecting the dynamic nature of the gut microbiome in early life (**Supplementary Table S2**). Of the seven pairs of twins enrolled in the LEGACY study, only two pairs shared the same stool microbiome cluster at 10 days of life. At 3 months, four of five pairs of twins (two lost to follow-up) shared the same microbiome cluster.

To confirm the lack of effect of maternal vaginal microbiome composition, we searched for taxa present in infant stool microbiomes at both timepoints which were significantly associated with each of the five canonical maternal CSTs. After correcting for multiple testing, MaAsLin2 identified two taxa- *Lactobacillus gasseri* and *Actinomyces vaccimaxillae*- which were significantly associated with infants born to mothers from CST II (*L. gasseri*-dominant vaginal microbiomes) at 10 days of life (**Supplementary File S3**). However, this association did not hold when comparing the relative abundances of these taxa across stool microbiomes stratified by maternal CST (**Supplementary Figure S4**, *P >*0.137, all comparisons). On the contrary, this is likely the result of differences in the distribution of samples where the abundance of these two taxa is extremely low, but not zero, among CST groups. In 3-month-old infants, a single taxon, *Hungatella hathewayi*, was significantly associated with maternal CST II, though only 267 reads of this taxon were detected across 10 samples in the entire dataset, making this observation unreliable.

Given the assumption that maternal microbiome exposure drives differences in infant stool microbiomes between vaginally and C/S-delivered infants, we also assessed whether these differences could be seen in the LEGACY cohort. For 10-day-old infant stool microbiomes, principal component analysis (PCA) showed varying degrees of separation by birth mode, but appreciable overlap of all groups. However, many vaginally-delivered infants clustered separately from caesarean-born infants (**Supplementary Figure 5A**, upper-right quadrant), and significant differences in microbiome composition by birth mode were detected for all pairwise comparisons (*P <*0.01). Scores across the first principal component, where most separation occurred at 10 days of life, were higher in vaginally-delivered infants compared to both elective and emergency C/S infants (**Supplementary Figure 5B**; *P <*0.0001) and were also higher in infants delivered by emergency rather than elective C/S (*P <*0.01). At 3-months of life, compositional differences in infant stool microbiomes were vastly reduced, with high amounts of overlap among all birth modes (**Supplementary Figure 5C**). Despite this, differences between vaginally delivered infants and both emergency and elective C/S deliveries remained statistically significant (*P <*0.05, both comparisons). No differences in alpha diversity were observed at either timepoint (**Supplementary Figures 5D, E**, *P >*0.99, all comparisons).

### Intrapartum antibiotic administration confounds microbiome differences attributed to delivery mode

**Figure 4:**
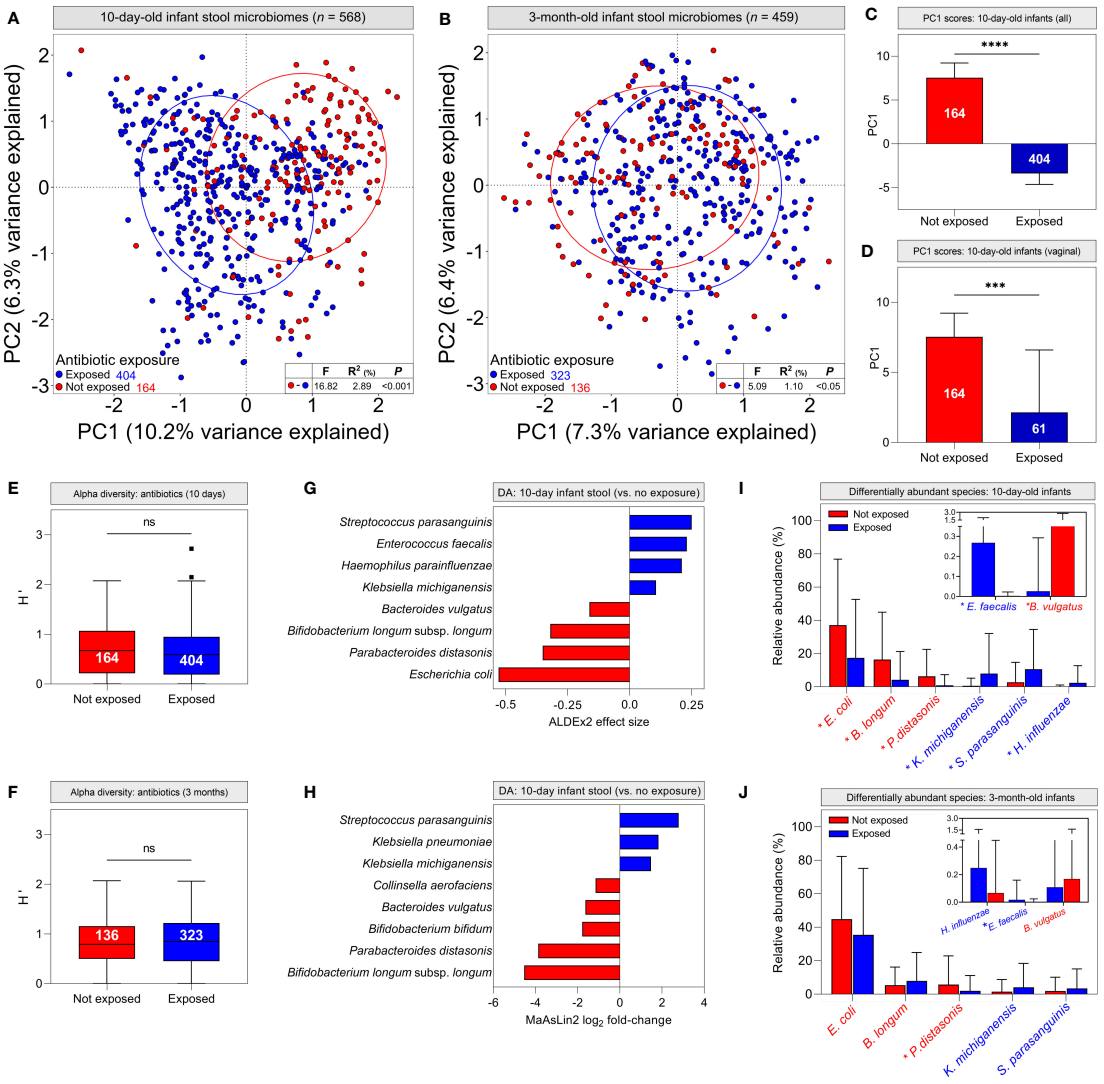
Intrapartum antibiotic exposure confounds infant stool microbiome differences commonly attributed to delivery mode: **(A, B)** Principal component analysis of *cpn*60 stool microbiome profiles grouped by antibiotic exposure for 10-day (**A**, pairwise PERMANOVA, *P <*0.001) and 3-month (**B**, *P <*0.05) -old infants. **(C, D)** Median scores across the first principal component for all 10-day-old infant stool microbiomes **(C)** and those delivered vaginally **(D)**, grouped by antibiotic exposure (bars indicate 95% CI). **(E, F)** Alpha diversity of stool microbiomes from 10-day **(E)** and 3-month **(F)** -old infants (bars indicate 1.5x IQR) (**C–F**, Mann-Whitney U, *** *P <*0.001, **** *P <*0.0001, ns, not significant). **(G, H)** Differentially abundant taxa in 10-day-old infant stool microbiomes as determined by ALDEx2 **(G)** and MaAsLin2 **(H)**. Bar colour indicates antibiotic exposure. **(I, J)** Mean relative abundances ( ± s.d.) of differentially abundant taxa in 10-day **(I)** and 3-month **(J)** -old infant stool microbiomes. Asterisks indicate significantly different mean abundance (FDR-corrected Mann-Whitney U test).

We next attempted to identify other clinical factors that could explain differences in infant stool microbiomes commonly attributed to birth mode, including breastfeeding modality, intrapartum antibiotics, maternal ethnicity, infant sex and chorioamnionitis. Hierarchical clustering suggested that intrapartum administration of antibiotics to mothers was associated with specific stool microbiome clusters at 10 days of life (**Supplementary Figure S6**). Stratifying infant stool microbiomes by maternal antibiotic administration during labour and delivery for any reason (e.g., caesarean delivery, GBS colonisation, suspected chorioamnionitis, etc.), PCA showed significant clustering of 10-day-old infant stool microbiomes based on antibiotic exposure (**Figure 4A**, *P <*0.001), which was less evident, albeit still statistically significant at 3 months (**Figure 4B**, *P <*0.05). PC1 scores for 10-day-old stool microbiomes were significantly lower in infants exposed to antibiotics when considering all infant stool microbiomes but also when restricting analysis to vaginally-delivered infants only (**Figures 4C, D**; *P <*0.0001 and <0.001, respectively). The similarity of these data to analyses grouped by delivery mode implies that delivery mode alone does not account for differences between infant stool microbiomes. To explore this further, we investigated the independent effects of delivery mode (comparing elective C/S infants with vaginally-delivered infants with antibiotic exposure) and intrapartum antibiotics (vaginally delivered infants with and without antibiotic exposure) on 10-day-old infant stool microbiomes. Both factors independently affected microbiome composition to a degree (**Supplementary Figure S7**; delivery mode, *P <*0.05; antibiotics, *P <*0.001).

Although alpha diversity did not differ by antibiotic exposure (**Figures 4E, F**; *P* = 0.156 and 0.642, respectively), differential abundance analysis identified species driving antibiotic-mediated microbiome differences. In 10-day-old infants, *Klebsiella michiganensis*, *Haemophilus parainfluenzae*, *Enterococcus faecalis* and *Streptococcus parasanguinis* were significantly more abundant in antibiotic-exposed infants, whereas *Escherichia. coli*, *Parabacteroides distasonis*, *Bacteroides vulgatus*, and *Bifidobacterium longum* subsp. *longum* were overrepresented in unexposed infants (**Figure 4G**; **Supplementary File S3**). These findings were supported by generalised linear modelling, with MaAsLin2 demonstrating associations between antibiotic exposure and many of the same species in infant stool microbiomes (**Figure 4H**; **Supplementary File S3**). Additionally, *Klebsiella pneumoniae* was associated with perinatal antibiotic exposure, while *Bifidobacterium bifidum* and *Collinsella aerofaciens* were associated with a lack thereof. When grouping infant stool microbiomes by delivery mode, both ALDEx2 and MaAsLin2 identified many of the same differentially abundant species as above when comparing vaginal and elective C/S deliveries; however, differences between emergency C/S and vaginal deliveries were more subtle. (**Supplementary Figure S8**). At 3 months, *P. distasonis* was the only differentially abundant taxon defined by ALDEx2 and was overrepresented in unexposed/vaginally-delivered infants, while MaAsLin2 identified an association only between elective C/S and *Enterococcus faecalis*. (**Supplementary Figure S8**; **Supplementary File S3**). Differential abundance data were corroborated by comparing relative abundances of these taxa between antibiotic exposure groups. All abundances were significantly different between groups in 10-day-old infants, but only *P. distasonis* and *E. faecalis* exhibited significantly different abundances at 3 months (**Figures 4I, J**).

**Figure 5:**
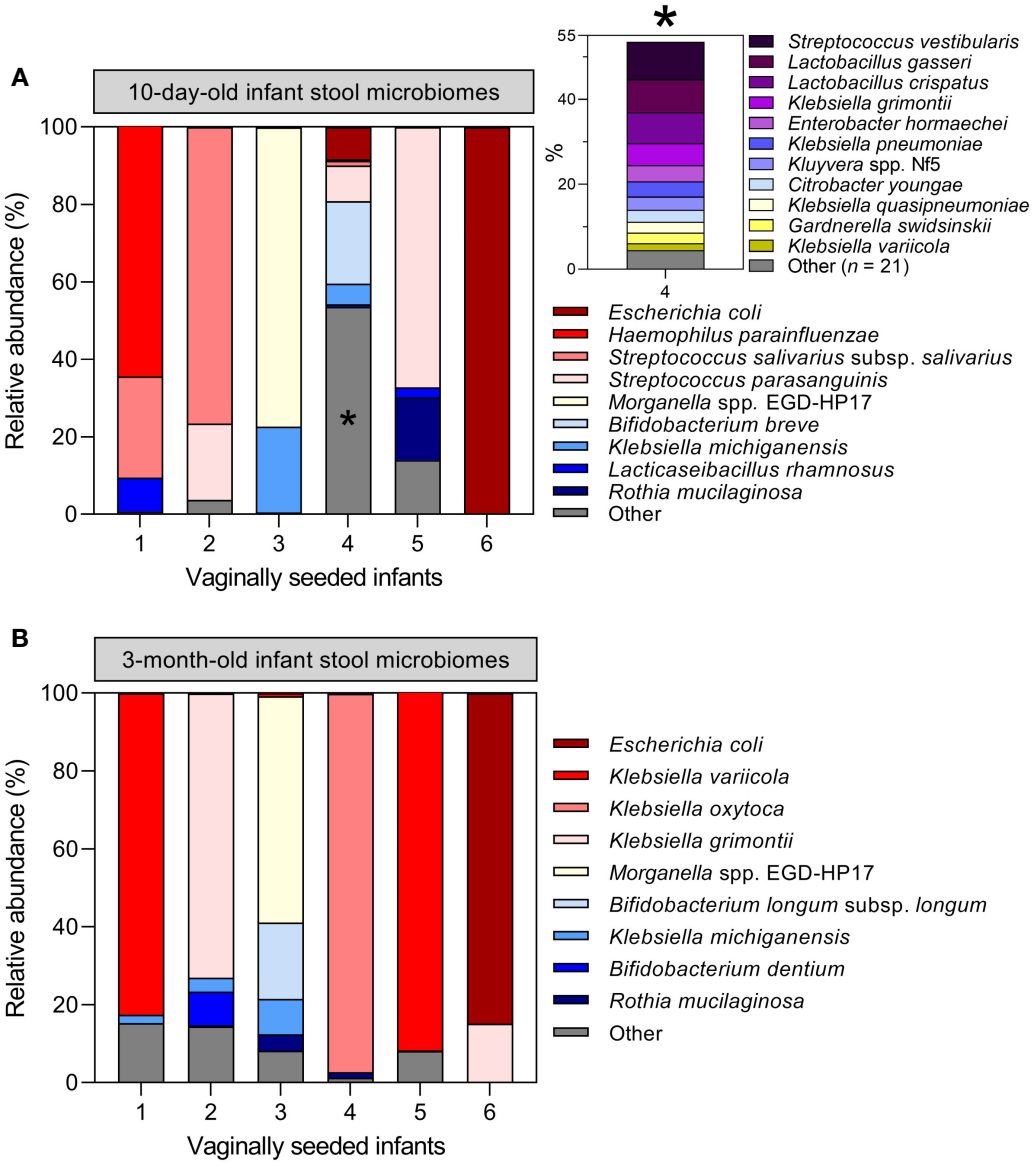
Vaginal seeding does not appear to restore the stool microbiome of C/S-delivered infants: Microbiome profiles of the six infants in the LEGACY cohort receiving supplementary maternal vaginal microbes by post-partum vaginal seeding at 10 days **(A)** and 3 months **(B)** of life. Inset plot in **(A)** represents 53.6% of the total *cpn*60 microbiome profile of infant 4 collapsed into ‘Other’, denoted by an asterisk *.

Assessment of maternal microbiome distribution among infant stool microbiome clusters by antibiotic exposure rather than birth mode did not alter the prior conclusion that the maternal vaginal microbiome is not predictive of infant stool microbiome composition (**Supplementary Figure S9**). Finally, six infants in the LEGACY cohort underwent vaginal seeding: a practice whereby maternal vaginal secretions are transferred to CS-delivered infants in an effort to correct birth mode-related dysbiosis. Therefore, we examined their stool microbiome profiles to determine if the procedure had restored taxa associated with vaginally-delivered infants. Only 1/6 infants undergoing vaginal seeding showed an abundance of taxa associated with vaginal delivery at either timepoint: the remaining 5/6 were dominated by taxa associated with C/S (**Figure 5**).

## Discussion

In one of the largest paired mother-infant cohorts to date, we demonstrated that maternal vaginal microbiome composition does not adequately predict the composition of the infant stool microbiome at 10 days or 3 months of life, regardless of delivery mode. Furthermore, major differences in infant stool microbiome composition which are commonly attributed to delivery mode (specifically a lack of exposure to the maternal vaginal microbiome) are explained at least in part by intrapartum antibiotic administration, confounding the potential effect of delivery mode.

Maternal vaginal microbiomes defined in this study were typical of the vaginal environment and reflected the results of prior studies (Albert et al., 2015; Freitas et al., 2017; Freitas et al., 2018; Wells et al., 2020) (low diversity, *Lactobacillus*-dominant), though the larger number of participants resulted in the separation of CST IV into twelve distinct clusters, each dominated by organisms typically associated with bacterial vaginosis. Infant stool microbiome profiles were similarly of low complexity, and with few exceptions, exhibited dominance of a single species in contrast to the highly diverse and species-rich gut communities of adults or older children (Yatsunenko et al., 2012). However, there was a high degree of heterogeneity in the species defining each stool cluster, particularly at 10 days of life.

Contrary to early reports of infant gut microbiota, we did not detect an abundance of *Lactobacillus* spp. in stool microbiomes of infants regardless of birth mode. We did, however, observe significant compositional differences by mode of delivery in early life, including between elective and emergency C/S deliveries. However, all individuals in the LEGACY cohort who underwent caesarean section received intravenous antibiotics (mostly cefazolin; **Supplementary Table S1**) as well as 70 who delivered vaginally (28.3%) after receiving antibiotic prophylaxis, (primarily penicillin for Group B streptococcal prophylaxis), highlighting intrapartum antibiotic exposure as a potential confounder of this relationship. Several previous studies identifying delivery mode as a mediator of infant stool microbiome composition either do not consider this confounder, or do not make clear the extent to which data have been adjusted for it (Dominguez-Bello et al., 2010; Bäckhed et al., 2015; Brumbaugh et al., 2016; Hill et al., 2017). In contrast, Reyman and colleagues explicitly addressed this issue in a study of 120 infants by administering antibiotic prophylaxis after clamping of the umbilical cord and found that delivery mode exerted a clear effect on infant stool microbiomes independent of antibiotic exposure (Reyman et al., 2019). However, administration of antibiotic prophylaxis prior to skin incision for C/S is widespread in clinical practice due to the improvement in post-surgical infection rates (van Schalkwyk and Van Eyk, 2017), therefore the effect of antibiotics on infant microbiomes cannot be discounted. While we observed a similar, independent effect of delivery mode on infant microbiomes, we also found an independent effect of intrapartum antibiotic use in vaginally-delivered infant stool microbiomes in addition to significant differences in ordination across the first principal component (increased *E. coli*, *Bifidobacterium* spp., *P. distasonis*). Similar findings have been reported by others studying the impact of intrapartum antibiotics (Azad et al., 2016; Bokulich et al., 2016; Yassour et al., 2016); therefore, while delivery mode may contribute to early life microbiome differences, it is often confounded by maternal antibiotic exposure during labour and delivery.

We identified three taxa in infant stool microbiomes significantly associated with maternal CST II; however, these taxa comprised relatively little of the total relative abundance of stool microbiomes when they were present (<1%). We attribute this finding to differences in the distribution of non-zero relative abundances between maternal CST groups and maintain that the maternal vaginal microbiome does not influence early-life stool microbiome composition in infants. Species that we identified as significantly different between antibiotic-exposed and -unexposed infants at 10 days of life overlapped well with taxa identified as significantly different between C/S and vaginally delivered infants, respectively. *Klebsiella* spp (Reyman et al., 2019; Shao et al., 2019)., *Enterococcus (* Azad et al., 2016; Reyman et al., 2019; Shao et al., 2019; Song et al., 2021) (spp. and specifically, *E. faecalis*), and *Streptococcus parasanguinis (* Shao et al., 2019) have all been reported as significantly overrepresented in stool microbiomes of C/S-delivered infants. Likewise, *Escherichia coli (* Bäckhed et al., 2015; Stewart et al., 2018; Reyman et al., 2019; Shao et al., 2019), *Parabacteroides* spp (Bäckhed et al., 2015; Bokulich et al., 2016; Hill et al., 2017; Reyman et al., 2019; Shao et al., 2019). (and *P. distasonis*), *Bacteroides* spp (Bäckhed et al., 2015; Azad et al., 2016; Bokulich et al., 2016; Dominguez-Bello et al., 2016; Hill et al., 2017; Stewart et al., 2018; Reyman et al., 2019; Shao et al., 2019)., and *Bifidobacterium (* Stewart et al., 2018; Reyman et al., 2019; Shao et al., 2019; Song et al., 2021) (spp. plus *B. longum*) have all been identified as overrepresented taxa in vaginally-delivered infants. There are conflicting reports on the persistence of delivery mode or antibiotic-associated microbiome differences throughout the initial year of life, ranging from no difference after 6 weeks, to detectable differences at 1 year (Azad et al., 2016; Chu et al., 2017; Busi et al., 2021; Song et al., 2021). Notably, more recent large scale-studies including ours, demonstrate convergence of vaginally- and C/S-delivered infant stool microbiomes within the first few months of life (Stewart et al., 2018; Shao et al., 2019; Song et al., 2021).

In contrast to other studies, members of the genus *Bacteroides* were not abundant in the majority of infant stool microbiomes from the LEGACY cohort. This may be due to the choice of molecular barcoding gene (*cpn*60 in the present study vs.16S rRNA hypervariable regions in almost all aforementioned studies), different sampling timepoints, and the plethora of different DNA extraction kits, all of which can affect microbiome composition considerably (Videnska et al., 2019; Soriano-Lerma et al., 2020). Despite this, *Bacteroides vulgatus* and *Bacteroides uniformis* were still identified as key organisms contributing to the differences between infant stool microbiomes at 10 days of life, highlighting the importance of this genus in the developing neonatal microbiome.

The lack of effect of maternal vaginal microbiome composition on the developing neonatal stool microbiome suggests the need to focus on factors impacting its development, including antibiotic exposure. In addition, the inability of vaginal seeding to ‘restore’ stool microbiomes of C/S-delivered infants in the small subset undergoing the procedure, as demonstrated by our data, supports the lack of validity behind the practice of vaginal seeding. Initial work on the efficacy of vaginal seeding showed a failure to restore the *Bacteroides* signature often observed in vaginally delivered infants and that anal swab microbiomes from seeded infants were more often classified as originating from a C/S-delivered infant rather than a vaginally delivered infant (Dominguez-Bello et al., 2016). Recent trials examining the effect of post-natal vaginal seeding on microbiome development in C/S-delivered infants have been contradictory. Song and colleagues reported a small shift in the composition of rectal swab microbiomes from vaginally seeded infants born by C/S towards those of vaginally-delivered infants across the first months of life (Song et al., 2021). However, Wilson et al. failed to find any impact of vaginal seeding on stool microbiomes in a randomised controlled trial from C/S-delivered infants, in which stool microbiomes from seeded and placebo groups both exhibited differences compared to vaginally-delivered infants at 1 month of life (Wilson et al., 2021). While larger trials are still ongoing, any effect of vaginal seeding appears to be transient and only evident immediately after birth prior to the impact of breastfeeding and other environmental exposures.

Similarly, data assessing the impact of vaginal microbiota at birth on infant stool microbiome development are limited: Dominguez-Bello and co-workers reported that rectal swab microbiomes of newborn infants closely resembled that of the maternal vagina in four vaginally delivered infants (Dominguez-Bello et al., 2010). Comparable findings were recorded by Chu et al. (2017), although both groups collected infant samples within 24 hours of birth and neither study was specifically designed to investigate the effect of vaginal microbiome composition. However, given that maternal vaginal microbiome composition does not influence that of the infant stool, we suggest that the impact of transferring a mother’s vaginal microbiome, if any, is likely to be limited.

The lack of influence exerted by the maternal vaginal microbiome on infant stool microbiome development underscores the much larger contributions of other maternal microbiomes, such as those of the breast milk and maternal gut. Breastfeeding is associated with higher abundances of *Bifidobacterium* spp. in infant stool within the first weeks and months of life (Ho et al., 2018) and metagenomic data on strain-specific transmission indicate vertical transfer of maternal faecal strains to the early infant gut. Identical strains from the genera *Ruminococcus*, *Bifidobacterium*, *Bacteroides* and members of the Clostridiales order have been identified in mother-infant pairs by multiple independent groups (Yassour et al., 2016; Asnicar et al., 2017; Ferretti et al., 2018; Wilson et al., 2021), particularly in the early days and weeks of life. Conversely, while strain transmission from the maternal vagina to infant gut has also been reported, this appears to be less common (Ferretti et al., 2018; Wilson et al., 2021). Together with our work, these data suggest more focus should be placed on maternal sources other than the vaginal microbiome when investigating the origins and development of infant microbiomes.

Our study is among the largest to characterise infant stool microbiomes within the first days of life and to directly investigate the influence of the maternal vaginal microbiome on neonatal stool microbiome development. Furthermore, sequencing of the *cpn*60 universal barcode enabled characterisation of microbiomes from the LEGACY cohort at superior resolution compared to the 16S rRNA gene (often limited to genus-level resolution), owing to greater sequence heterogeneity between closely related species (Links et al., 2012). Pairwise comparisons of *cpn*60 and 16S rRNA gene sequences in a collection of 1,349 genome sequences determined that the median sequence similarity of *cpn*60 universal target regions to the next closest sequence was significantly lower than that of the 16S rRNA gene hypervariable regions, ranging from 82-92% for the former and 96-100% for the latter (Links et al., 2012). Likewise, a recent evaluation of cpnDB showed that median intra-genus sequence similarities of *cpn*60 were routinely below 90% (Vancuren and Hill, 2019), consistent with earlier literature demonstrating the higher level of resolution achieved when using this gene (Goh et al., 1996; Goh et al., 2000; Hill et al., 2006). Accordingly, application of *cpn*60 microbiome profiling in studies of the vaginal microbiome revealed previously unseen CSTs delineated by different *Gardnerella vaginalis* subgroups (Albert et al., 2015). The genus *Gardnerella* was recently emended to describe four named species of *Gardnerella (* Vaneechoutte et al., 2019) which correspond to the subgroups defined by *cpn*60 sequencing; however, these differences were not apparent based on sequence similarity of their 16S rRNA genes (Paramel Jayaprakash et al., 2012).

Our study has several limitations. Most individuals in the LEGACY cohort were white or Asian, resulting in a lower representation of black, indigenous and hispanic participants. Studies have repeatedly found a higher prevalence of *L. iners* and non-*Lactobacillus* dominant vaginal microbiomes (including *Gardnerella* spp. and *Atopobium vaginae*) in these groups compared to white individuals (Wells et al., 2020). Increased representation of such microbiomes in our study would have been helpful to increase the numbers of smaller vaginal microbiome clusters dominated by non-*Lactobacillus* species and maximise the generalisability of our findings. The distribution of ethnicities in the LEGACY study closely reflects that of the province where the study cohort was recruited, and the proportion of non-white participants was higher than in many other studies (Yassour et al., 2018; Maqsood et al., 2019). Furthermore, we did not formally compare infant stool microbiomes by breastfeeding modality; however, very few infants at either timepoint were exclusively formula-fed which precluded meaningful analysis, and there did not appear to be any difference in distribution of infant stool microbiome clusters by exclusive vs. non-exclusive breastfeeding. As part of the LEGACY project, breast milk samples and areola swabs were collected from mothers as part of the 10-day and 3-month postpartum visits, and analyses of these samples are ongoing.

Overall, we have shown in a large cohort of mother-infant pairs that exposure to the maternal vaginal microbiome during birth, as well as its composition, does not specifically influence the development of the infant stool microbiome in early infancy. Moreover, we have demonstrated that associations of delivery mode and infant stool microbiome composition appear to be impacted by exposure to intrapartum antibiotics and not primarily related to mode of delivery, which points to modifiable factors for future considerations.

## Data availability statement

All sequence data associated with this study were deposited in the NCBI Sequence Read Archive (BioProject PRJNA824125). Annotated R code describing reproducible analysis of sequencing data, processed feature tables containing nearest neighbour read counts, and microbiome cluster metadata are available to download from the online version of this article (**Supplementary Files S1–S3**).

## Ethics statement

The studies involving human participants were reviewed and approved by University of British Columbia Children’s and Women’s Health Centre Research Ethics Board, harmonized with partner boards in the Fraser Health Authority and Northern Health Authority (Certificate no. H17-02253). The patients/participants provided their written informed consent to participate in this study.

## Author contributions

DM devised the original concept of the work, and all authors contributed to study design. ZP and EM contributed to clinical data acquisition. JH and SD conceived and planned the experiments and SD conducted the experiments. SD performed the data analysis with help from AA. All authors contributed to interpretation of the results. SD wrote the first draft of the manuscript. ZP and DM wrote sections of the manuscript. All authors contributed to the article and approved the submitted version. 
