# Supplementary material for: Maternal vaginal microbiome composition does not affect development of the infant gut microbiome in early life
Source: Front Cell Infect Microbiol. 2023 Mar 30;13:1144254. doi: 10.3389/fcimb.2023.1144254 (PMC10097898; doi:10.3389/fcimb.2023.1144254)
Supplement: Supplementary file 4 [file Image_1.pdf]

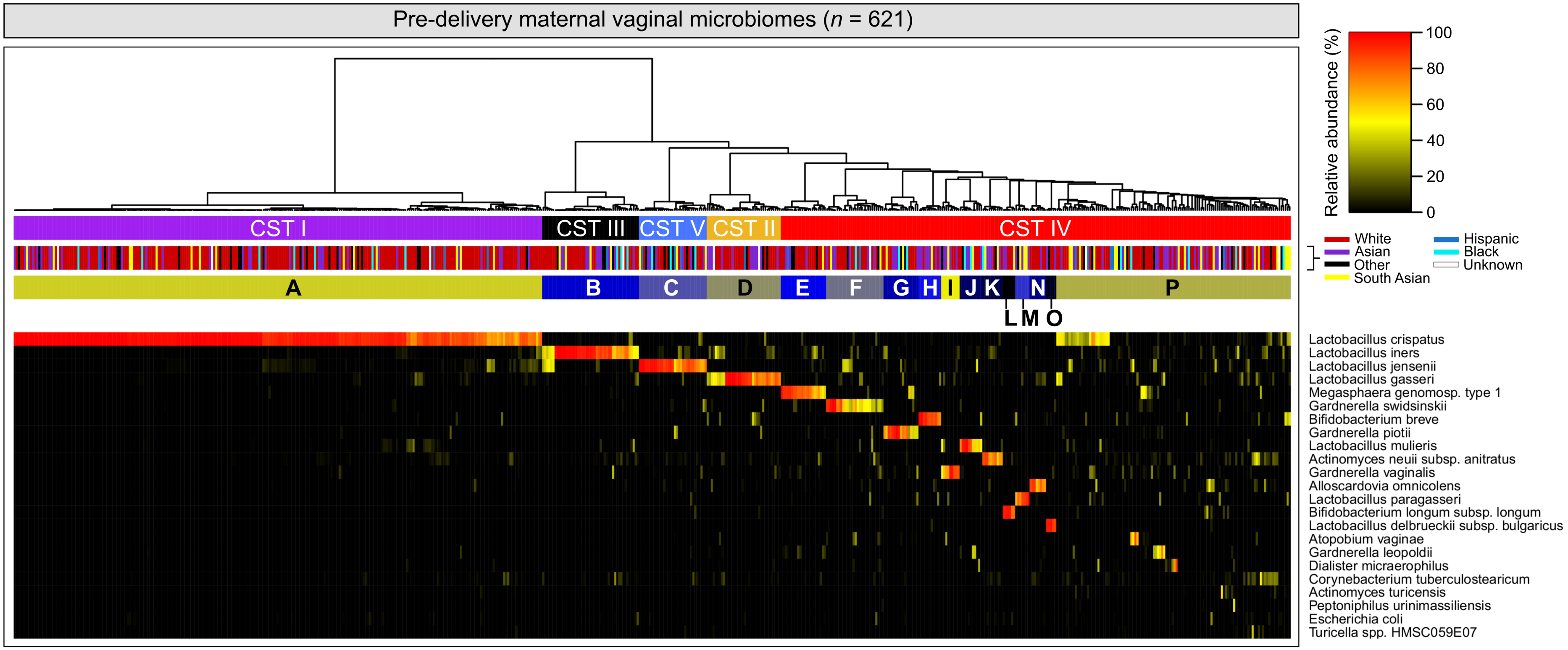

Supplementary Figure S1 Maternal vaginal microbiomes are dominated by one of several species of *Lactobacillus* or various anaerobic species: Hierarchical clustering of *cpn*60 vaginal microbiomes constructed from Euclidean distance matrices of proportional abundance data. Only select taxa shown. Top colour bar represents the five 'canonical' vaginal community state types (CSTs: I = *L. crispatus*, II = *L. gasseri*, III = *L. iners*, IV = various anaerobic species, V = *L. jensenii*), middle colour bar represents maternal ethnicity, bottom colour bar represents clusters defined by nbClust (left to right: Cluster A - Cluster P).
