# Supplementary material for: Maternal vaginal microbiome composition does not affect development of the infant gut microbiome in early life
Source: Front Cell Infect Microbiol. 2023 Mar 30;13:1144254. doi: 10.3389/fcimb.2023.1144254 (PMC10097898; doi:10.3389/fcimb.2023.1144254)
Supplement: Supplementary file 5 [file Image_2.pdf]

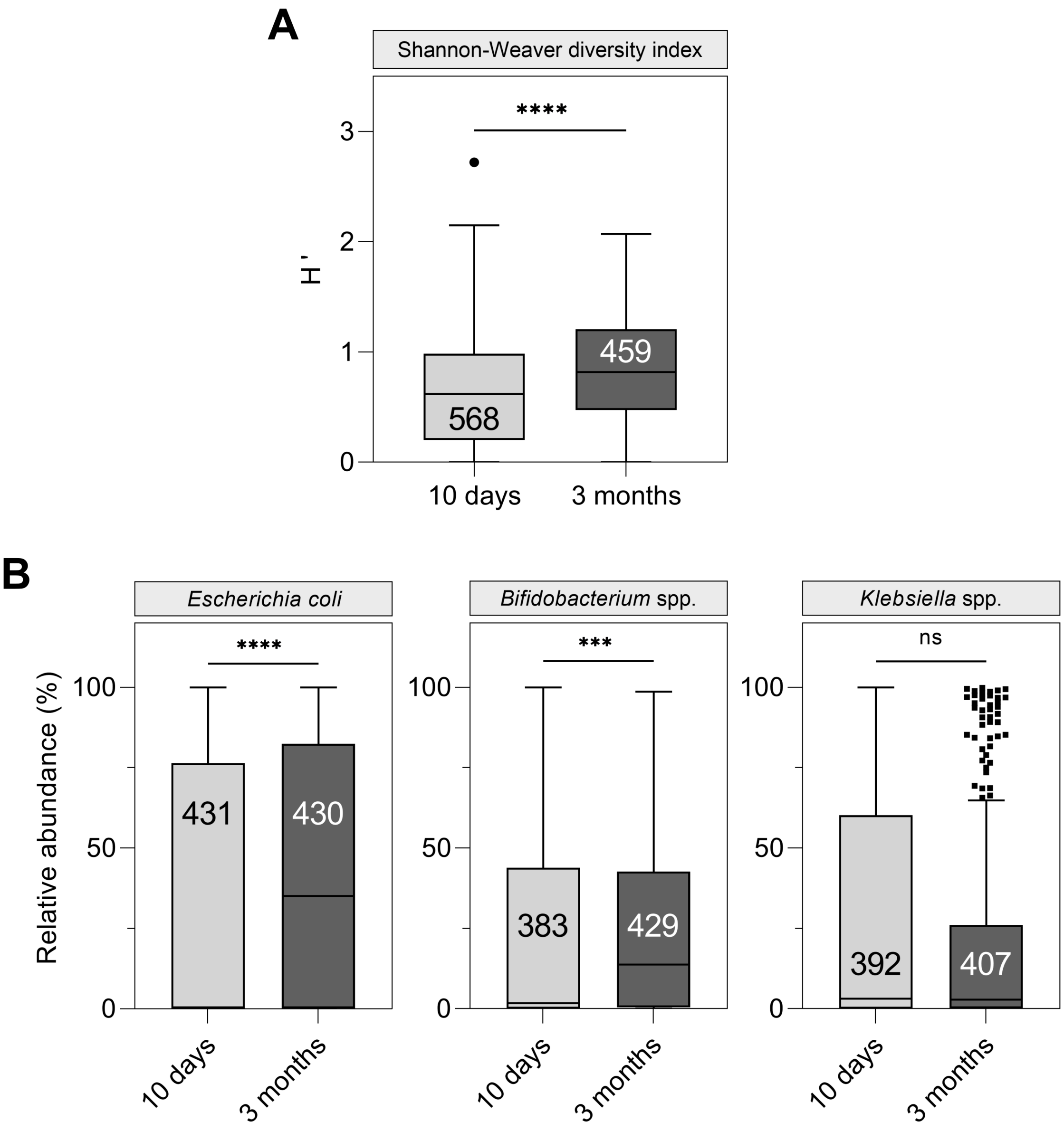

Supplementary Figure S2 Alpha diversity and relative abundances of *E. coli* and *Bifidobacterium* spp. in infant stool microbiomes increase over time: (**A**) Alpha diversity, measured by Shannon-Weaver index (H'), was calculated for infant stool microbiomes at 10-days and 3-months of life (bars indicate 1.5x IQR). (**B**) Boxplots of relative abundances of the most commonly detected taxa in infant stool microbiomes across time. Horizontal bar indicates median, Whiskers indicate 1.5x IQR. White text indicates number of samples. *** = P <0.001, **** = P <0.0001, ns = not significant (Mann-Whitney U).
