# Supplementary material for: Maternal vaginal microbiome composition does not affect development of the infant gut microbiome in early life
Source: Front Cell Infect Microbiol. 2023 Mar 30;13:1144254. doi: 10.3389/fcimb.2023.1144254 (PMC10097898; doi:10.3389/fcimb.2023.1144254)
Supplement: Supplementary file 6 [file Image_3.pdf]

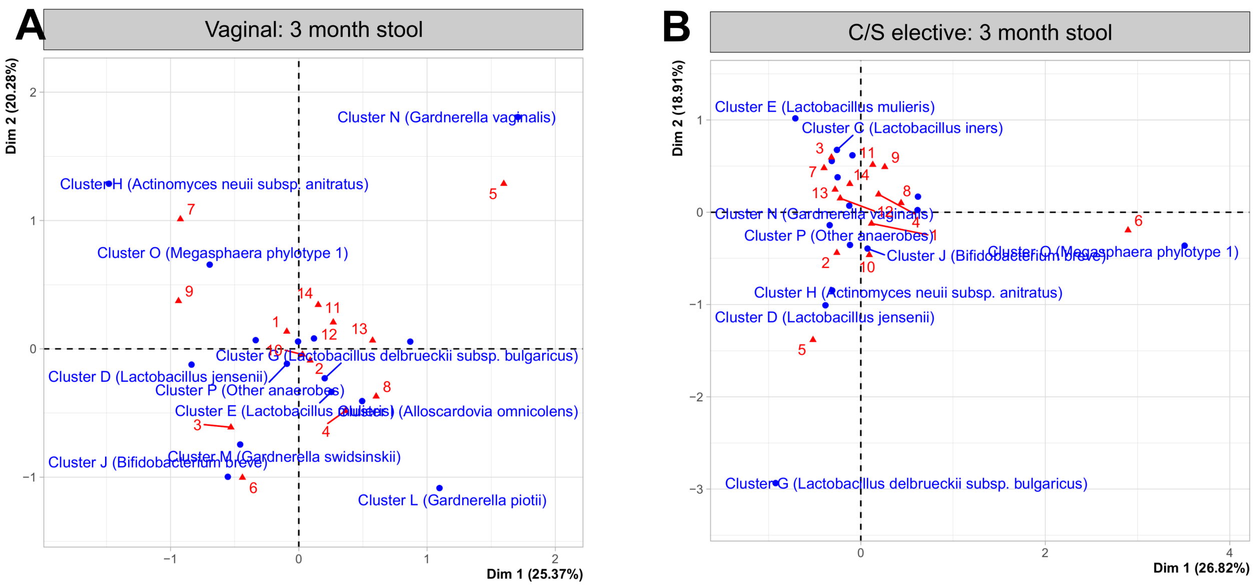

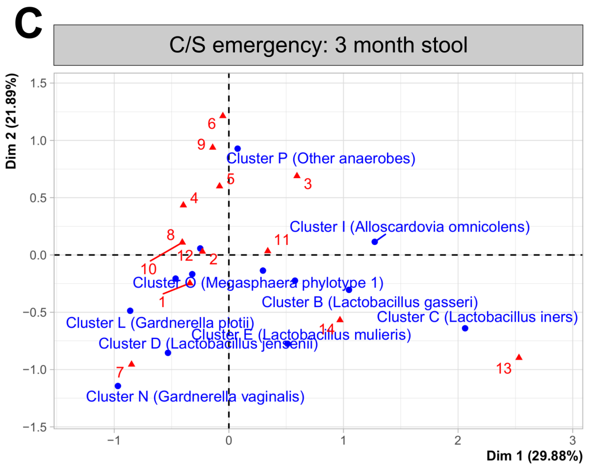

D

Time	*P* (Fisher's exact test)		
	Elective C/S	Emergency C/S	Vaginal
10 days	0.254	0.356	0.224
3 months	0.462	0.527	0.746

Supplementary Figure S3 Distribution of maternal vaginal microbiomes among infant stool clusters is not affected by delivery mode: Correspondence analysis of maternal vaginal microbiomes and infant stool clusters from 3-month-old infant stool microbiomes, grouped by vaginal delivery (**A**), elective caesarean (**B**) or emergency caesarean (**C**). *P* values from Fisher's exact test examining dfifferences in the distribution of maternal vaginal microbiome clusters among infant stool microbiome clusters (**D**) were corrected for multiple testing. Results are shown for analyses at 10 days and 3 months of life and stratified by delivery mode.
