# Supplementary material for: Maternal vaginal microbiome composition does not affect development of the infant gut microbiome in early life
Source: Front Cell Infect Microbiol. 2023 Mar 30;13:1144254. doi: 10.3389/fcimb.2023.1144254 (PMC10097898; doi:10.3389/fcimb.2023.1144254)
Supplement: Supplementary file 7 [file Image_4.pdf]

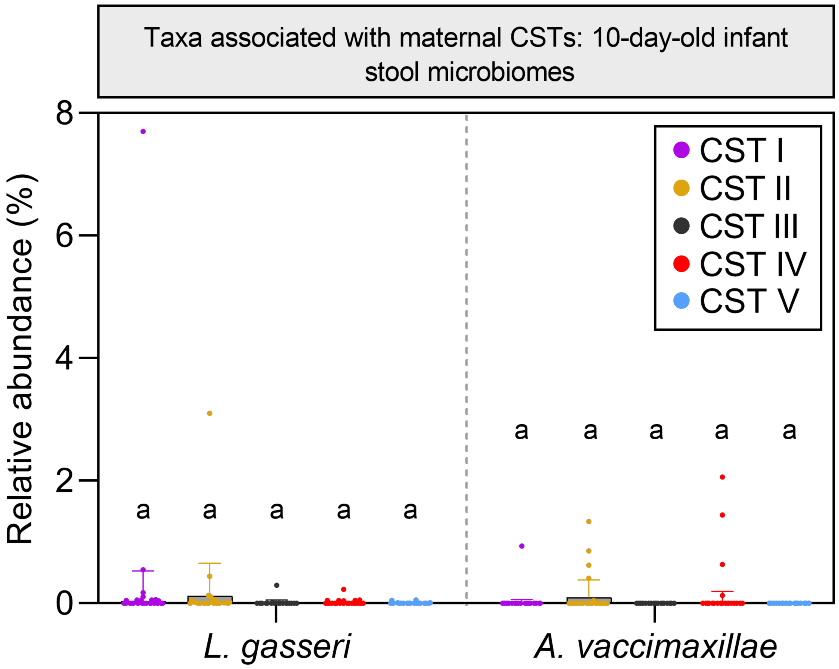

	L. gasseri					A. vaccimaxillae				
**No. samples**	245	34	40	220	29	245	34	40	220	29
**No. non-zero**	36	15	4	22	7	2	5	1	5	0
**Median abund.**	0.02	0.03	0.02	0.02	0.01	0.48	0.85	0.02	0.20	n/a

Supplementary Figure S4 Significant associations of specific taxa with CST II are not observed when comparing relative abundances in infant stool microbiomes: Relative abundances of the two taxa significantly associated with CST II (identified by MaAsLin2) are compared across 10-day-old infant stool microbiomes stratified by maternal CST. Total number of samples per maternal CST group, number of samples with a non-zero relative abundance taxa, and median relative abundance among samples with non-zero abundance are indicated for both taxa. Identical letters indicate no significant difference in median rank of relative abundance between groups (Kruskal-Wallis test, *P* >0.138 , all comparisons, adjusted for multiple comparisons)
