# Supplementary material for: Maternal vaginal microbiome composition does not affect development of the infant gut microbiome in early life
Source: Front Cell Infect Microbiol. 2023 Mar 30;13:1144254. doi: 10.3389/fcimb.2023.1144254 (PMC10097898; doi:10.3389/fcimb.2023.1144254)
Supplement: Supplementary file 8 [file Image_5.pdf]

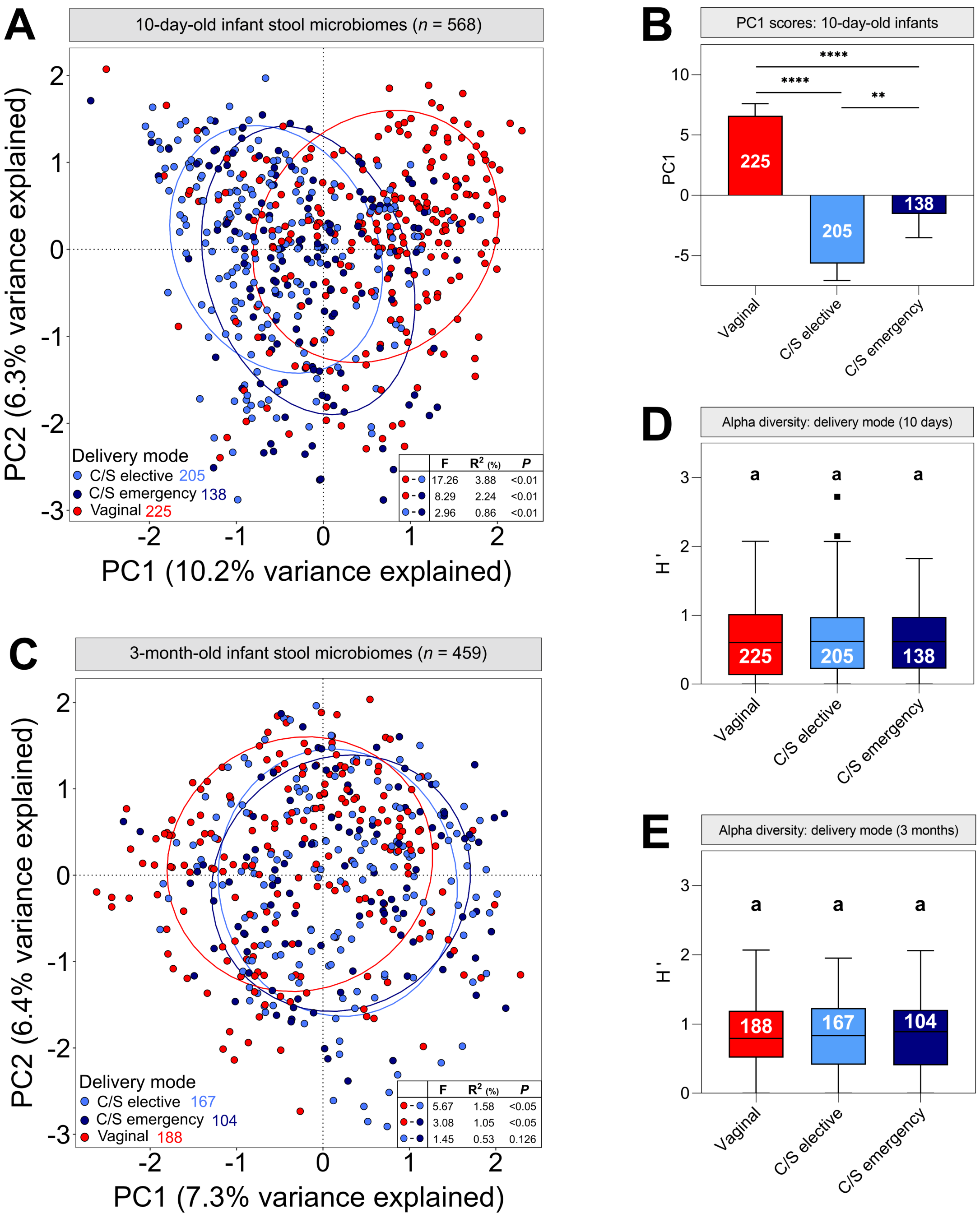

Supplementary Figure S5 Differences in infant stool microbiome by delivery mode are transient: (**A**) Principal component analysis (PCA) of *cpn*60 stool microbiome profiles from 10-day-old infants, grouped by delivery mode (pairwise PERMANOVA, *P* <0.01, all comparisons). (**B**) Median scores across the first principal component from 10-day-old infant stool microbiomes (bars indicate 95% CI, Kruskal-Wallis test w/ Dunn's correction, ** = *P* <0.01, **** = *P* <0.0001). (**C**) (PCA) of *cpn*60 stool microbiome profiles from 3-month-old infants, grouped by delivery mode (pairwise PERMANOVA, *P* <0.05, vaginal vs. both C/S). (**D&E**) Alpha diversity of stool microbiomes from 10-day (**D**) and 3-month (**E**) -old infants (bars indicate 1.5x IQR). Identical letters indicate no significant difference between groups (Kruskal-Wallis test w/ Dunn's correction).
