# Supplementary material for: Maternal vaginal microbiome composition does not affect development of the infant gut microbiome in early life
Source: Front Cell Infect Microbiol. 2023 Mar 30;13:1144254. doi: 10.3389/fcimb.2023.1144254 (PMC10097898; doi:10.3389/fcimb.2023.1144254)
Supplement: Supplementary file 9 [file Image_6.pdf]

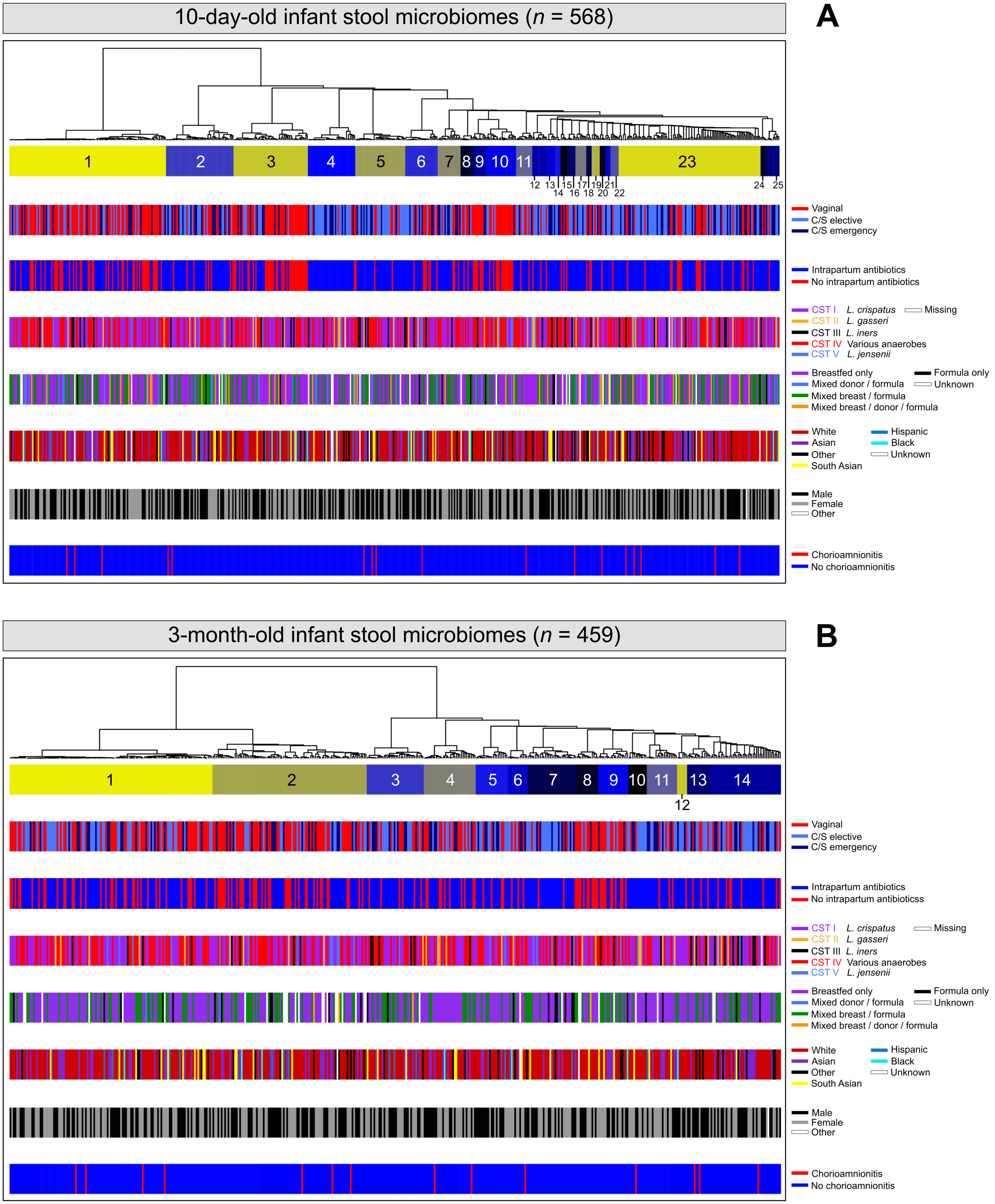

Supplementary Figure S6 Clinical factors associated with infant stool microbiome clusters: Hierarchical clustering of cpn60 infant stool microbiome clusters from 10-day (**A**) and 3-month (**B**) -old infants were constructed from Euclidean distance matrices of proportional abundance data. Colours bars from top to bottom: microbiome cluster, delivery mode, intrapartum antibiotic administration to mothers at labour and delivery, maternal vaginal CST, breastfeeding modality, maternal ethnicity, infant sex and maternal chorioamnionitis status.
