# Supplementary material for: Maternal vaginal microbiome composition does not affect development of the infant gut microbiome in early life
Source: Front Cell Infect Microbiol. 2023 Mar 30;13:1144254. doi: 10.3389/fcimb.2023.1144254 (PMC10097898; doi:10.3389/fcimb.2023.1144254)
Supplement: Supplementary file 10 [file Image_7.pdf]

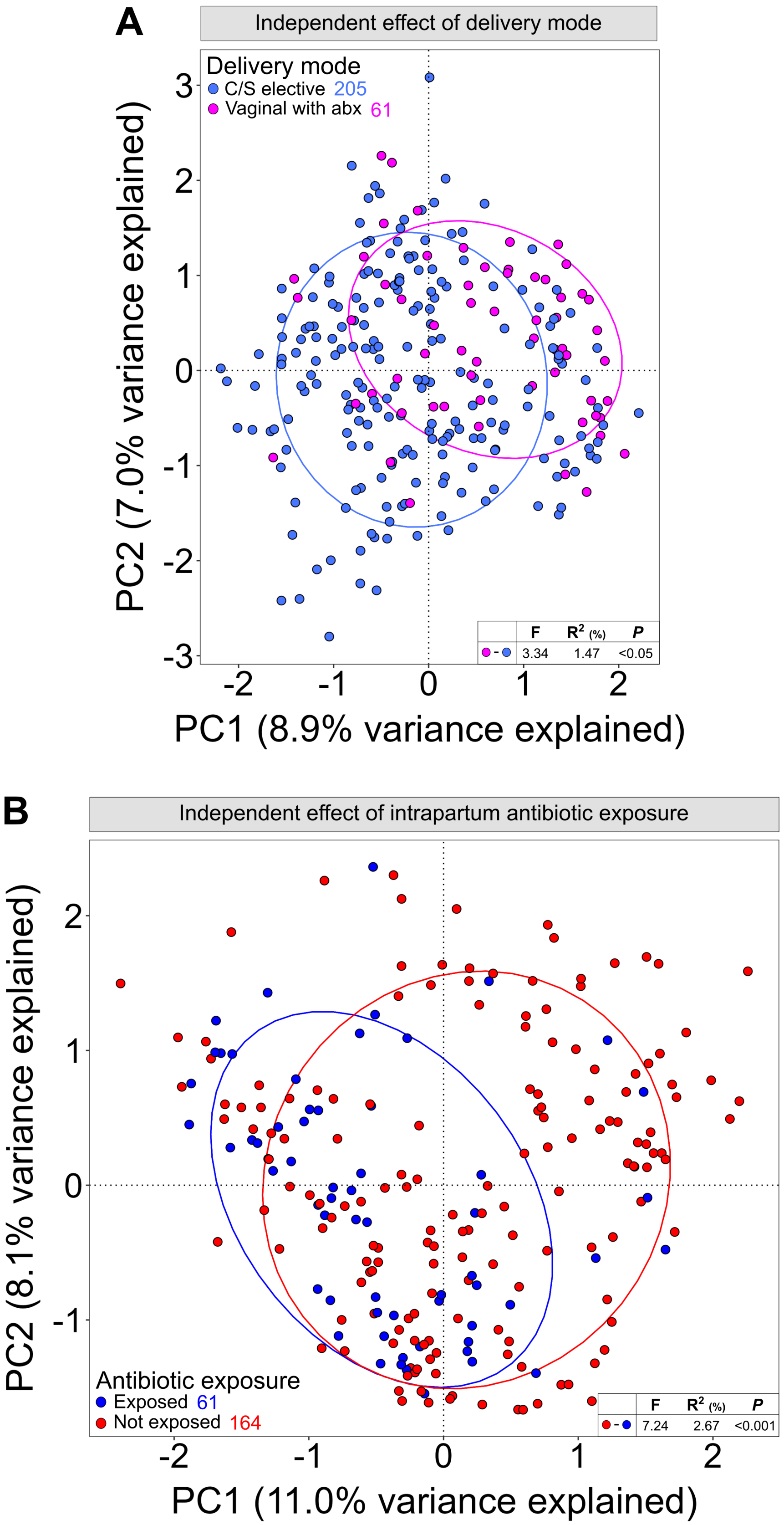

Supplementary Figure S7 Independent effect of delivery mode and intrapartum antibiotics: (**A**) Principal component analysis of *cpn*60 stool microbiome profiles from 10-day-old infants delivered vaginally with antibiotic exposure and by elective C/S (F = 3.34, R^2 =1.47, P <0.05). Differences are assumed to be due to delivery mode alone. (**B**) PCA of *cpn*60 stool microbiome profiles from 10-day-old vaginally delivered infants with and without intrapartum antibiotic exposure (F = 7.24, R^2 = 2.67, P <0.001). Differences are assumed to be due to antibiotics alone.
