# Supplementary material for: Maternal vaginal microbiome composition does not affect development of the infant gut microbiome in early life
Source: Front Cell Infect Microbiol. 2023 Mar 30;13:1144254. doi: 10.3389/fcimb.2023.1144254 (PMC10097898; doi:10.3389/fcimb.2023.1144254)
Supplement: Supplementary file 11 [file Image_8.pdf]

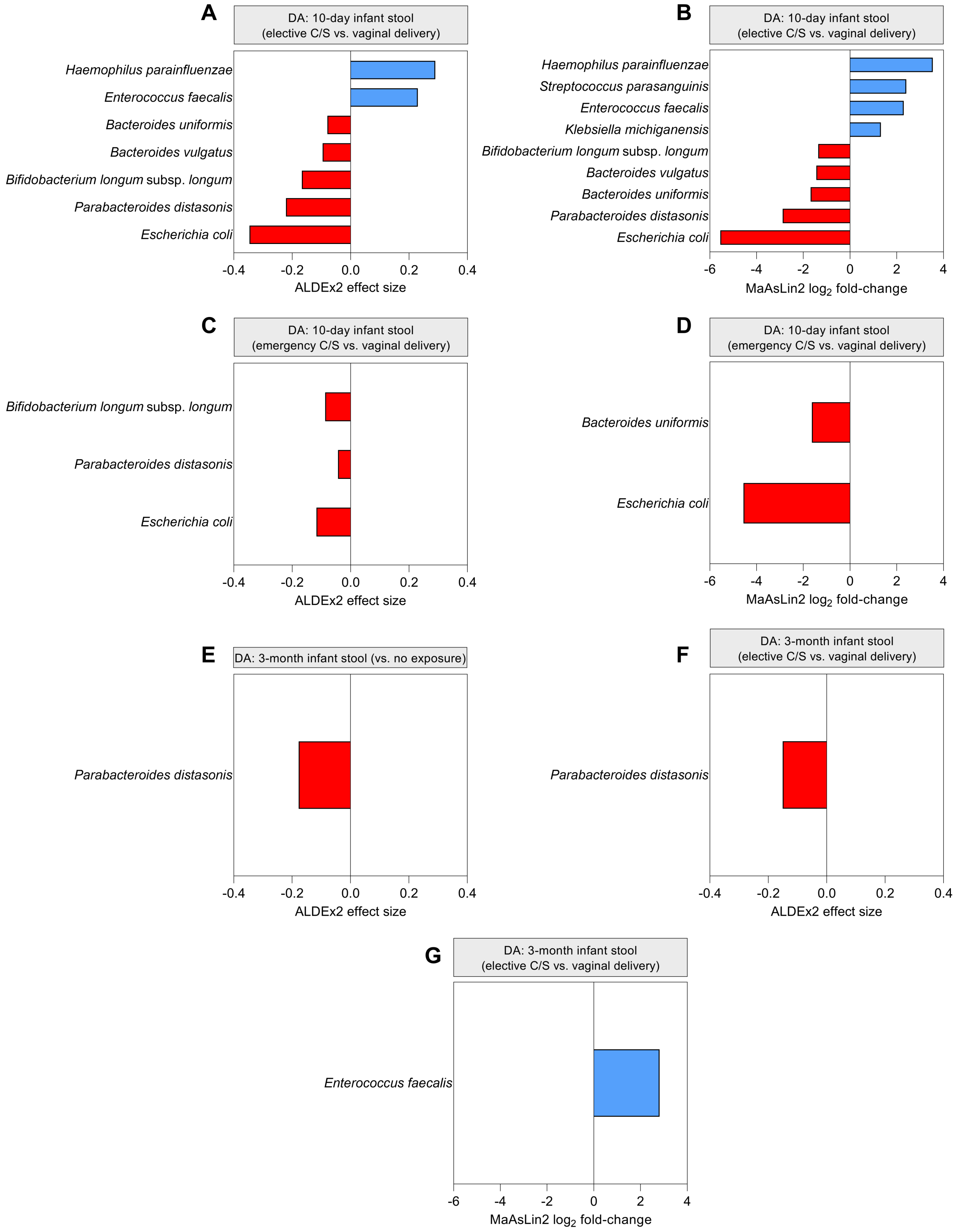


**Supplementary Figure S8 Similar species are differentially abundant when grouping infant stool microbiomes by delivery mode or perinatal antibiotic exposure:** Differentially abundant species in stool microbiomes from 10-day (**A**-**D**) and 3-month (**E**-**G**) -old infants were defined by ALDEx2 (**A**,**C**,**E**,**F**) and MaAsLin2 (**B**,**D**,**G**). Infants were grouped either by delivery mode, or by perinatal antibiotic exposure. Red bars indicate overrepresentation in vaginally-delivered or unexposed infants and light blue bars indicate overrepresentation in infants delivered by elective caesarean section.
