# Supplementary material for: Maternal vaginal microbiome composition does not affect development of the infant gut microbiome in early life
Source: Front Cell Infect Microbiol. 2023 Mar 30;13:1144254. doi: 10.3389/fcimb.2023.1144254 (PMC10097898; doi:10.3389/fcimb.2023.1144254)
Supplement: Supplementary file 12 [file Image_9.pdf]

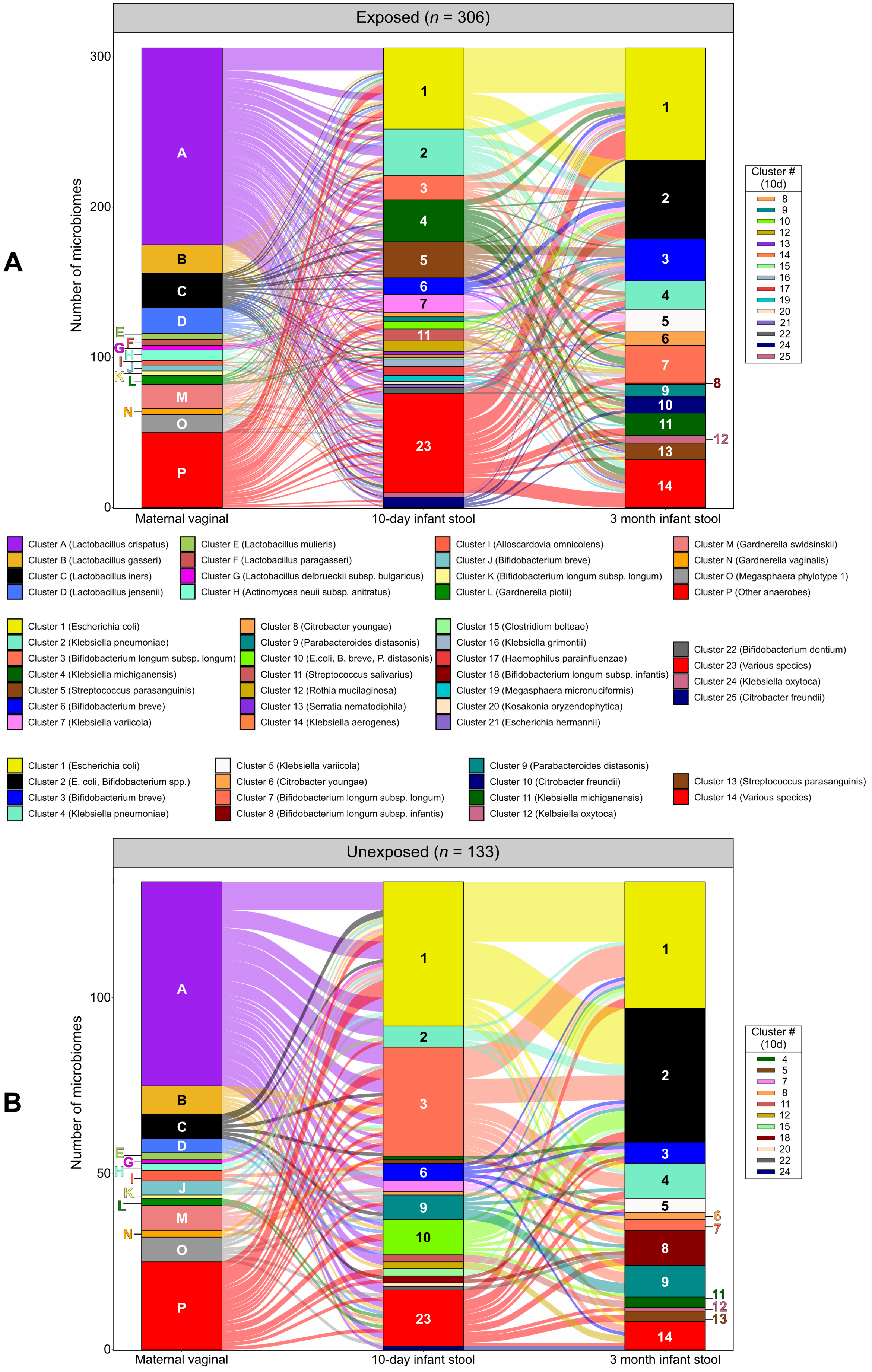

Supplementary Figure S9 Lack of association between maternal vaginal and infant stool microbiome clusters is not affected by antibiotic exposure: Alluvial flow of mother-infant dyads from pre-delivery maternal microbiome clusters to 3-month-old infant stool microbiome clusters for infants exposed to antibiotics during labour and delivery (**A**) and those not exposed (**B**). No evident patterns are present and vaginal microbiomes are still distributed among infant stool clusters according to their frequency.
