# Supplementary material for: Maternal vaginal microbiome composition does not affect development of the infant gut microbiome in early life
Source: Front Cell Infect Microbiol. 2023 Mar 30;13:1144254. doi: 10.3389/fcimb.2023.1144254 (PMC10097898; doi:10.3389/fcimb.2023.1144254)
Supplement: Supplementary file 13 [file Table_1.pdf]

Supplementary Table 1: Characteristics of the LEGACY study cohort (623 participants and their infants), stratified by mode of delivery. Parentheses indicate row proportions.

	Mode of Delivery [a]				
	**Total (*n* = 623)**	**Vaginal (*n* = 247)**	**Emergency C/S (*n* = 155)**	**Elective C/S (*n* = 221)**	***P* value [b]**
**Maternal characteristics**					
**Age at delivery, years**					
Mean ± SD	34.6 ± 4.3	33.8 ± 4.4	34.7 ± 4.5	35.4 ± 3.9	**<0.001**
20-29	73 (11.7%)	42 (17.0%)	16 (10.3%)	15 (6.8%)	**0.012**
30-39	470 (75.4%)	181 (73.3%)	119 (76.8%)	170 (76.9%)	
40-49	79 (12.7%)	24 (9.7%)	20 (12.9%)	35 (15.8%)	
50-59	1 (0.2%)	0 (0%)	0 (0%)	1 (0.5%)	
**Ethnicity**					
White/Caucasian	341 (54.7%)	154 (62.3%)	71 (45.8%)	116 (52.5%)	0.109
Asian	132 (21.2%)	43 (17.4%)	34 (21.9%)	55 (24.9%)	
South Asian	52 (8.3%)	16 (6.5%)	21 (13.5%)	15 (6.8%)	
Mixed Ethnicity	25 (4.0%)	9 (3.6%)	7 (4.5%)	9 (4.1%)	
Other	19 (3.0%)	5 (2.0%)	5 (3.2%)	9 (4.1%)	
Hispanic	14 (2.2%)	4 (1.6%)	6 (3.9%)	4 (1.8%)	
Indigenous/Aboriginal/First Nations/Metis/Inuit	10 (1.8%)	4 (1.6%)	2 (1.3%)	4 (1.8%)	
African/Caribbean/Black	6 (1.0%)	4 (1.6%)	2 (1.3%)	0 (0%)	
Unknown	24 (3.9%)	8 (3.2%)	7 (4.5%)	9 (4.1%)	
**BMI pre- or early pregnancy**					
Mean ± SD	24.6 ± 5.2	24.9 ± 5.6	25.1 ± 5.6	23.9 ± 4.3	0.056
<18.5 (Underweight)	14 (2.2%)	4 (1.6%)	3 (1.9%)	7 (3.2%)	
18.5-24.9 (Normal)	367 (58.9%)	151 (61.1%)	89 (57.4%)	127 (57.5%)	
25-29.9 (Overweight)	140 (22.5%)	54 (21.9%)	29 (18.7%)	57 (25.8%)	
>=30 (Obese)	71 (11.4%)	31 (12.6%)	25 (16.1%)	15 (6.8%)	
Unknown	31 (5.0%)	7 (2.8%)	9 (5.8%)	15 (6.8%)	
**Gravidity**					
1	254 (40.8%)	109 (44.1%)	88 (56.8%)	57 (25.8%)	**<0.001**
2	206 (33.1%)	81 (32.8%)	33 (21.3%)	92 (41.6%)	
3+	161 (25.8%)	57 (23.1%)	34 (21.9%)	70 (31.7%)	
Unknown	2 (0.3%)	0 (0%)	0 (0%)	2 (0.9%)	
**Parity**					
0	365 (58.6%)	152 (61.5%)	127 (81.9%)	86 (38.9%)	**<0.001**
1	216 (34.7%)	82 (33.2%)	22 (14.2%)	112 (50.7%)	
2+	39 (6.3%)	13 (5.3%)	6 (3.9%)	20 (9.0%)	
Unknown	3 (0.5%)	0 (0%)	0 (0%)	3 (1.4%)	
**Group B *Streptococcus***					
Negative	425 (68.2%)	184 (74.5%)	110 (71.0%)	131 (59.3%)	**<0.001**
Positive	133 (21.3%)	59 (23.9%)	37 (23.9%)	37 (16.7%)	
Not tested	60 (9.6%)	2 (0.8%)	7 (4.5%)	51 (23.1%)	
Unknown	5 (0.8%)	2 (0.8%)	1 (0.6%)	2 (0.9%)	
**Hypertension of pregnancy**					
No	557 (89.4%)	222 (89.9%)	125 (80.6%)	210 (95.0%)	**<0.001**
Yes	65 (10.4%)	25 (10.1%)	30 (19.4%)	10 (4.5%)	
Unknown	1 (0.2%)	0 (0%)	0 (0%)	1 (0.5%)	

	(cont.) Mode of Delivery [a]				
	Total (*n* = 623)	Vaginal (*n* = 247)	Emergency C/S (*n* = 155)	Elective C/S (*n* = 221)	*P* value [b]
**Gestational diabetes (GDM)**					
No	485 (77.8%)	192 (77.7%)	119 (76.8%)	174 (78.7%)	
Yes	103 (16.5)	39 (15.8%)	29 (18.7%)	35 (15.8%)	0.860
Unknown	35 (5.6)	16 (6.5%)	7 (4.5%)	12 (5.4%)	
**Control of GDM**					
Insulin	50 (48.5%)	23 (59.0%)	12 (41.4%)	15(42.9%)	
Diet	52 (50.5%)	16 (41.0%)	16 (55.2%)	20 (57.1%)	0.278
Unknown	1 (1.0%)	0 (0%)	1 (0.3%)	0 (0%)	
**Delivery characteristics**					
**Delivery location**					
Hospital	602 (96.6%)	226 (91.5%)	155 (100%)	221 (100%)	**<0.001**
Home	21 (3.4%)	21 (8.5%)	0 (0%)	0 (0%)	
**Intrapartum antibiotics**					
No	177 (28.4%)	177 (71.7%)	0 (0%)	0 (0%)	**<0.001**
Yes	446 (71.6%)	70 (28.3%)	155 (100%)	221 (100%)	
**Type of antibiotic [c,d]**					
Cefazolin	387 (86.8%)	23 (32.9%)	152 (98.1%)	213 (96.4%)	
Penicillin G	84 (18.8%)	50 (71.4%)	34 (21.9%)	0	
Metronidazole	38 (8.5%)	9 (12.9%)	26 (16.8%)	3 (1.4%)	
Clindamycin	10 (2.2%)	2 (2.9%)	3 (1.9%)	6 (2.7%)	
Other	3 (0.7%)	2 (2.9%)	1 (0.6%)	0	
Unknown	5 (1.1%)	1 (1.5%)	0	2 (0.9%)	
**Chorioamnionitis [e]**					
No	603 (96.8%)	238 (96.4%)	144 (92.9%)	221 (100%)	**<0.001**
Yes	20 (3.2%)	9 (3.6%)	11 (7.1%)	0 (0%)	
**Duration of ROM**					
<18hrs	566 (90.9%)	221 (89.5%)	124 (80.0%)	221 (100%)	
>=18hrs	37 (5.9%)	23 (9.3%)	14 (9.0%)	0 (0%)	**<0.001**
Unknown	20 (3.2%)	3 (1.2%)	17 (11.0%)	0 (0%)	
**Number infants delivered**					
Singleton	614 (98.6%)	246 (99.6%)	154 (99.4%)	214 (96.8%)	**0.008**
Twins	9 (1.4%)	1 (0.4%)	1 (0.6%)	7 (3.2%)	
**Infant characteristics (n=632)**					
**Sex**					
Female	307 (48.6%)	129 (52.0%)	72 (46.2%)	106 (46.5%)	
Male	324 (51.3%)	119 (48.0%)	83 (53.2%)	122 (53.5%)	0.292
Other	1 (0.2%)	0 (0%)	1 (0.6%)	0 (0%)	
**Birth Weight**					
Mean ± SD (g)	3450 ± 499	3445 ± 466	3443 ± 540	3459 ± 507	0.944
**Gestational Age at Birth**					
Mean ± SD (weeks)	39.5 ± 1.2	39.7 ± 1.3	39.6 ± 1.4	39.1 ± 0.8	**<0.001**

[a] C/S; caesarean section

[b] *P* values from χ^2 test

[c] Parentheses indicate proportion of 446 participants receiving intrapartum antibiotics

[d] Column totals exceed 100% due to some participants receiving multiple different antibiotics

[e] Including suspected chorioamnionitis diagnosed based on clinical presentation
