# Supplementary material for: Maternal vaginal microbiome composition does not affect development of the infant gut microbiome in early life
Source: Front Cell Infect Microbiol. 2023 Mar 30;13:1144254. doi: 10.3389/fcimb.2023.1144254 (PMC10097898; doi:10.3389/fcimb.2023.1144254)
Supplement: Supplementary file 14 [file Table_2.pdf]

Supplementary Table S2: Stability of infant gut microbiomes between 10 days and 3 months of life split by delivery mode. Infant microbiomes were classed as stable if they belonged to clusters sharing the same dominant organism at 10-day and 3-month timepoints (e.g., stable infants dominated by *Bifidobacterium breve* belong to cluster 6 at 10 days and cluster 3 at 3 months). ND indicates no stable microbiomes.

**10 day stool cluster [a]**	**No. of 'stable' microbiomes**	**Delivery mode**		
		**C/S elective**	**C/S emergency**	**Vaginal**
All	88/360	27/120	20/78	41/162
**1** (*Escherichia coli*)	48/96	12/21	8/15	28/60
**2** (*Klebsiella pneumoniae*)	9/38	3/15	2/12	4/11
**3** (*Bifidobacterium longum* subsp. *longum*)	6/47	1/6	3/8	2/33
**4** (*Klebsiella michiganensis*)	5/29	2/18	3/8	0/3
**5** (*Streptococcus parasanguinis*)	3/25	2/14	1/7	0/4
**6** (*Bifidobacterium breve*)	2/16	1/7	0/3	1/6
**7** (*Klebsiella variicola*)	5/16	3/5	2/7	0/4
**8** (*Citrobacter youngae*)	3/4	1/2	ND	2/2
**9** (*Parabacteroides distasonis*)	4/10	ND	1/2	3/8
**10** (*E. coli*, *B. breve*, *P. distasonis*)	0/15	0/3	ND	0/12
**11** (*Streptococcus salivarius*)	0/10	0/5	0/1	0/4
**12** (*Rothia mucilaginosa*)	0/9	0/6	0/1	0/2
**13** (*Serratia nematodophila*)	0/2	0/1	0/1	ND
**14** (*Klebsiella aerogenes*)	0/2	ND	0/2	ND
**15** (*Clostridium boltae*)	0/3	ND	0/1	0/2
**16** (*Klebsiella grimontii*)	0/5	0/2	0/1	0/2
**17** (*Haemophilus parainfluenzae*)	0/6	0/5	0/1	ND
**18** (*Bifidobacterium longum* subsp. *infantis*)	1/2	ND	ND	1/2
**19** (*Megasphaera micronuciformis*)	0/4	0/1	0/1	0/2
**20** (*Kosakonia oryzendophytica*)	0/3	0/1	0/1	0/1
**21** (*Escherichia hermanii*)	0/2	0/1	0/1	ND
**22** (*Bifidobacterium dentium*)	0/5	0/3	0/1	0/1
**24** (*Klebsiella oxytoca*)	0/3	ND	0/1	0/2
**25** (*Citrobacter freundii*)	2/8	2/4	0/3	0/1

[a] Excluding 82 infants whose microbiome profile at 10 days belonged to cluster 23 (various species)
